# Modelling the effects of cell-to-cell variability on the output of interconnected gene networks in bacterial populations

**DOI:** 10.1186/1752-0509-9-S3-S6

**Published:** 2015-06-01

**Authors:** Nicolò Politi, Lorenzo Pasotti, Susanna Zucca, Paolo Magni

**Affiliations:** 1 Dipartimento di Ingegneria Industriale e dell'Informazione, Università degli Studi di Pavia, via Ferrata 5, Pavia, Italy; 2 Centro di Ingegneria Tissutale, Università degli Studi di Pavia, via Ferrata 5, Pavia, Italy

## Abstract

**Background:**

The interconnection of quantitatively characterized biological devices may lead to composite systems with apparently unpredictable behaviour. Context-dependent variability of biological parts has been investigated in several studies, measuring its entity and identifying the factors contributing to variability. Such studies rely on the experimental analysis of model systems, by quantifying reporter genes via population or single-cell approaches. However, cell-to-cell variability is not commonly included in predictability analyses, thus relying on predictive models trained and tested on central tendency values. This work aims to study *in silico *the effects of cell-to-cell variability on the population-averaged output of interconnected biological circuits.

**Methods:**

The steady-state deterministic transfer function of individual devices was described by Hill equations and lognormal synthetic noise was applied to their output. Two- and three-module networks were studied, where individual devices implemented inducible/repressible functions. The single-cell output of such networks was simulated as a function of noise entity; their population-averaged output was computed and used to investigate the expected variability in transfer function identification. The study was extended by testing different noise models, module logic, intrinsic/extrinsic noise proportions and network configurations.

**Results:**

First, the transfer function of an individual module was identified from simulated data of a two-module network. The estimated parameter variability among different noise entities was limited (14%), while a larger difference was observed (up to 62%) when estimated and true parameters were compared. Thus, low-variability parameter estimates can be obtained for different noise entities, although deviating from the true parameters, whose measurement requires noise knowledge. Second, the black-box input-output function of a two/three-module network was predicted from the knowledge of the transfer function of individual modules, identified in the presence of noise. Estimates variability was low (16%); however, differences up to 68% were observed by simulating a typical experimental study where the predictions obtained above were compared to network outputs generated in the presence of noise. Network predictions can, thus, deviate from real outputs when modules are characterized and re-used in different noise contexts.

**Conclusions:**

The adopted approach can support predictability studies in synthetic biology by distinguishing between actual unpredictability and contribution of noise and by guiding researchers in the design of suitable experimental measurement for gene networks.

## Background

The bottom-up engineering of living systems exhibiting predictable functions is one of the main goals of synthetic biology [1-3]. This approach relies on the modularity of the used components [4-7] or the predictability of system function upon parts interconnection, environmental change or genetic context variation [8,9]. A reliable biological system design process will open the door to the full exploitation of synthetic biology's potential, which will benefit many application fields, like medicine and bioenergy, via the construction of customized systems for new drugs or fuel biosynthesis [10,11]. However, the interconnection of quantitatively characterized biological parts may lead to composite systems with apparently unpredictable behaviour, that is, the output cannot be intuitively gathered from the available knowledge of sub-parts [5,6,12]. Basic research studies on ad-hoc constructed model systems have been conducted to elucidate the entity of context-dependent variability of biological parts and devices and, ultimately, to identify the factors contributing to this variability [6,13-19]. In such studies, components like promoters or simple inducible/repressible devices were individually characterized and used to engineer genetic networks of increasing complexity, including transcriptional regulator cascades, feedback-controlled systems or networks mimicking logic functions. The comparison between the experimental output of interconnected circuits and mathematical model predictions has been useful to evaluate the predictability boundaries of biological components when re-used to engineer diverse systems [20]. Moreover, some of the factors affecting system modularity have been found, such as DNA sequences that might enhance or decrease transcriptional activity when placed upstream or downstream of promoters [6,15] or retroactivity effects due to the presence of DNA binding sites in interconnected modules [8,21].

Context-dependent variability studies have also led to the development of specific genetic architectures, which significantly enhance the predictability of re-used components, such as insulation sequences for promoters [15] and bicistronic design (BCD) for ribosome binding sites (RBSs) [22].

In almost all the mentioned research studies fluorescent reporter proteins, like the Green Fluorescent Protein (GFP) and Red Fluorescent Protein (RFP), were adopted to experimentally measure the output of a genetic network *in vivo*. Population or single-cell approaches were used to quantify fluorescence levels: fluorometers or multiwell microplate readers were adopted to measure the average fluorescence in the cell population [6,15,18,19], while flow-cytometry was also commonly adopted to measure the fluorescence of each single cell in the population [13,16,17]. Much of our current knowledge of biology relies on population-average measurements, which can lead to incorrect conclusions when populations are not homogeneous or when inter-individual variability is crucial [23]. For this reason, single-cell approaches have widely spread to accurately analyze cell-to-cell variability in populations. However, even when single-cell analyses are carried out, fluorescence distributions among cells are not fully taken into account during the development, training and testing of predictive mathematical models of the network. For this reason, only central tendency measures of fluorescence are used, like arithmetic/geometric mean or median, thus using single-cell approaches as a quality check for measurements, with fluorescence histograms only exploited as a control of cell homogeneity [16,24]. In particular, arithmetic mean can be used to compare data obtained from flow cytometry and fluorometers/microplate reader acquisitions [25]. On the other hand, only a few studies fully exploited cell-to-cell variability to build up predictive models able to describe the behaviour of genetic devices re-used in different configurations. For instance, Guido et al. used the Gillespie stochastic algorithm to capture the single-cell behaviour of a synthetic promoter (inducible by isopropyl β-D-1-thiogalactopyranoside - IPTG - and repressible by arabinose) and the trained model was successfully used to predict the output distribution among recombinant cells bearing a feedback-controlled network including this promoter, as well as cell-to-cell variability quantitative changes upon plasmid copy number modifications [13].

Different fluorescence levels among cells in the same population can be attributable to noise [26]. Noise is one of the main features characterizing gene expression in living cells and it can be considered as a highly important source of phenotypic variation in cell populations [23,26]. It is important to consider the stochastic nature of chemical reactions, which can lead to important phenomena regarding biological processes such as development, particularly when dealing with molecular species that are present in low or very low copy number inside cells. [26,27]. There are two main aspects of noise that can interact: an "intrinsic" component, due to the stochastic nature of the biochemical processes linked to gene expression itself, and an "extrinsic" one, which is caused by fluctuations in cellular species such as per cell concentrations of polymerases [26,28-30]. Noise in synthetic biological circuits has been widely studied to elucidate the entity of its intrinsic and extrinsic components in different contexts [26], characterize its propagation through interconnected networks [31-33], investigate its contributions in transcription and translation processes [29], study its effects in complex biological functions like oscillatory networks composed of transcriptional regulators [34,35] and, finally, even to exploit it for the discovery of unknown regulatory motifs via dynamic correlation in time-course data [36,37]. Noise has also been already used to explain apparently unpredictable outputs of genetic circuits that cannot be described by deterministic models [38].

This work aims to study *in silico *the effects of cell-to-cell variability on the population-averaged output of interconnected biological circuits. In particular, we aim to investigate if central tendency measures, obtained via population-based or single-cell approaches, are suitable to study the input-output behaviour of individual and interconnected devices, or, conversely, if the entity of cell-to-cell variability needs to be included to accurately describe these input-output functions. Considering the available experimental data of several studies where the interconnection of modules results in apparently unpredictable circuits, this study can elucidate if some observed inconsistencies might be caused by cell-to-cell variability. The computational analysis of several systems and variability models can support the experiment design of interconnected networks.

## Methods

### Genetic circuits topology

**Figure 1:**
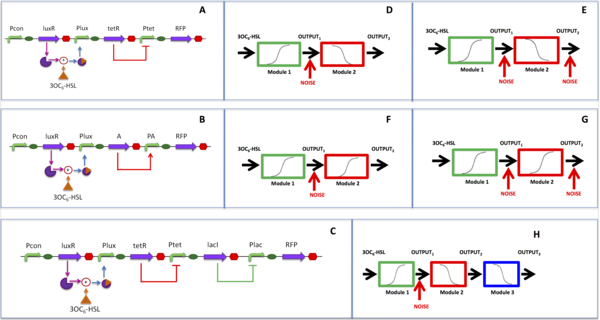
**Gene networks used in this work**. The three main transcriptional regulatory cascades are illustrated. A-C) Genetic circuit structure. The underlying inducible and repressible mechanisms are reported: in the presence of LuxR, 3OC_6_-HSL activates Plux; TetR and LacI, encoded by the tetR and lacI genes, respectively, repress Ptet and Plac; the A protein, encoded by the A gene, activates PA. Curved arrows represent promoters, straight arrows represent genes, ovals represent RBSs and octagons represent transcriptional terminators. Pcon is a constitutive promoter. D-H) Block schemes for the genetic circuits, where, for each module, the steady-state transfer function is qualitatively described by reporting its input-output trend. Module 1 is the LuxR/Plux-based 3OC_6_-HSL-inducible device (panels D-H); Module 2 is TetR/Ptet NOT gate in panels D, E, H, while it is the YES gate in panels F, G; for some experiments reported in the Results section, the TetR/Ptet NOT gate was replaced by a LacI/Plac NOT gate, which has the same repressible logic and this configuration is not shown in this figure; Module 3 is the LacI/Plac NOT gate (panel H). Noise is applied to the output of Module OUTPUT_1 _(panels D, F) or to both OUTPUT_1 _and OUTPUT_2 _(panels E, G, H).

The genetic networks studied in this work are shown in Figure 1. They are transcriptional regulatory cascades commonly used in synthetic biology studies [6,13,17,21]. These networks are composed of inducible and repressible devices with a reporter gene downstream, such as RFP, to measure the circuit output. In particular, an N-3-oxohexanoyl-L-homoserine lactone (3OC_6_-HSL)-inducible device is always used as the input module of the network; it is composed of a constitutive expression cassette for the LuxR protein, which activates the Plux promoter in the presence of 3OC_6_-HSL in a concentration-dependent fashion. A genetic NOT gate is considered as downstream module, composed of a TetR protein expression cassette, driven by the upstream device promoter, and the Ptet promoter, which can be repressed by TetR in a concentration-dependent fashion. Another NOT gate device is also considered; its functioning is analogous to the TetR/Ptet system and it includes the LacI/Plac repressor-promoter pair. Finally, a genetic YES gate is considered, which is composed of an activator protein (herein called A) expression cassette, driven by the upstream device promoter, and a promoter (herein called PA) that is activated by A in a concentration-dependent fashion. While the lux, tet and lac systems are well-defined and literature parameters are used to model their functioning (see below), the A/PA system is mock and arbitrary parameters are used for it.

The paper is mainly focused on the network of Figure 1A, where the 3OC_6_-HSL-inducible device is interconnected to a TetR-/Ptet-based NOT gate. Two network modifications are also considered, where the NOT gate is replaced by a YES gate (Figure 1B) or where a LacI/Plac-based NOT gate device is interconnected downstream of the TetR/Ptet-based NOT gate (Figure 1C).

### *In silico *experiments

Assuming the modularity of biological parts [8,9], that is, the module parameters do not change when different units are interconnected, we aim to simulate experimental studies to evaluate the contribution of noise on the population-averaged network output. Two main investigations were performed, which are described below. The *in silico *experiments are carried out by considering the network at the steady-state and by using Hill equations to describe the transfer function of each individual module. The output of interconnected networks is obtained by serially propagating the output of Hill functions and noise is applied at different levels of the networks, as illustrated in Figure 1D-H.

#### Transfer function identification for a single module

The steady-state input-output transfer function of the TetR/Ptet-based NOT gate (or the YES gate) is measured by estimating its Hill function parameters from simulated data, generated by including noise obeying specified laws. In particular, 3OC_6_-HSL-inducible devices affected by different noise entities were used to drive the NOT gate over a range of input values. The input devices are assumed to provide identical population-averaged signals to drive the NOT gate, except for the noise entity affecting them. In an experimental framework, this is analogous to having a set of inducible devices whose population-averaged transfer function at the steady-state has been identified via central tendency measures and the model of noise affecting it is known. Previous studies were performed via a similar setup [6,39], where the transfer function of the NOT gate module was identified in the presence of different input modules, pre-characterized via central tendency measures, and different transfer curves were yielded for the NOT gate; since the different input modules used may have different noise characteristics, this study aims to evaluate the contribution of noise on the resulting difference of the measured transfer curves.

#### Input-output function identification for an interconnected network

The black-box input-output transfer function of interconnected networks (see Figure 1A-C) is studied for different noise entities. Population-averaged measures of the individual modules, in the presence of noise, are used to identify their transfer functions and to predict the input-output characteristics of the full network. Then, noise of different entities is applied to the individual modules and it is propagated throughout the network. Finally, the population-averaged measures of the network output are used to identify the black-box Hill function of the whole circuit and it is compared to the prediction obtained above. This study aims to evaluate the contribution of noise on the apparent unpredictability of composite biological systems obtained by interconnecting modules characterized via central tendency measures. The studied situation represents a crucial aspect in the synthetic biology world, where predictable systems are expected to be built from the knowledge of a set of individual parts [3,5].

### Steady-state transfer functions of the genetic modules

The steady-state input-output activity of all the modules is modelled by Hill equations, describing the synthesis rate per cell of the protein encoded by the downstream module, as a function of input molecule concentration [40-42]. In fact, even if all the considered modules are based on transcription, the synthesis (or translation) rate per cell of the downstream gene-encoded protein is assumed to be proportional to the transcription rate generated by the input device. This assumption is based on simple gene expression models, such as the ones described in [43,44].

All the investigated interconnected networks are studied at the steady-state.

The transfer function of the 3OC_6_-HSL-inducible system (Module 1, Figure 1D-H) is always modelled as:

(1) $$OUTPUT_{1}=\delta_{IN}+\frac{\alpha_{IN}}{1+{\left(\frac{k_{IN}}{3OC_{6}-HSL}\right)}^{\eta_{IN}}}$$

In Eq.1, the output of the device depends on 3OC_6_-HSL inducer concentration via four parameters describing its behaviour: *δ_IN _*is the basal activity of the device when no inducer is present, *δ_IN _*+*α_IN _*is the maximal synthesis rate of the downstream protein, *k_IN _*is the inducer concentration giving an output of $\delta_{IN}+\frac{\alpha_{IN}}{2}$, and *η_IN _*is the Hill coefficient. The *k_IN _*parameter is expressed in the same units as 3OC_6_-HSL (nM), the *η_IN _*parameter is dimensionless and the *δ_IN _*and *α_IN _*parameters are measured Relative Promoter Units (RPUs) [43], which are used to approximate the downstream protein synthesis rate per cell triggered by the device [6].

The function of the TetR-based NOT gate (Module 2, Figure 1D, E, H) is modelled as:

(2) $$OUTPUT_{2}=\delta_{OUT}+\frac{\alpha_{OUT}}{1+{\left(\frac{OUTPUT_{1}}{k_{OUT}}\right)}^{\eta_{OUT}}}$$

In Eq.2, OUTPUT_2 _is a decreasing function of OUTPUT_1 _and parameters have the same meaning as above. Although OUTPUT_1 _is the TetR synthesis rate per cell, it is used to approximate the intracellular concentration of TetR protein, assuming that it is proportional to its synthesis rate per cell [43]; in this case, the proportionality constant is included in the *k_OUT _*parameter.

When considering the three-module network, the function of the LacI-based NOT gate (Module 3, Figure 1H) is modelled as:

(3) $$OUTPUT_{3}=\delta_{OUT3}+\frac{\alpha_{OUT3}}{1+{\left(\frac{OUTPUT_{2}}{k_{OUT3}}\right)}^{\eta_{OUT3}}}$$

In Eq.3, all the parameters have the same meaning as in Eq.2.

The function of the YES gate (Module 2, Figure 1F,G) is modelled as:

(4) $$OUTPUT_{2}=\delta_{OUT}+\frac{\alpha_{OUT}}{1+{\left(\frac{k_{OUT}}{OUTPUT_{1}}\right)}^{\eta_{OUT}}}$$

In this case (Eq.3), OUTPUT_2 _is an increasing function of OUTPUT_1_, as opposed to Eq.2. In Figure 1D-H, the qualitative trends of the Hill functions for all the modules are reported. They can be considered as the deterministic transfer functions of all the devices, in absence of any stochastic effect, i.e., noise.

### Models of noise

Here, noise was assumed to affect protein synthesis rate per cell (see Figure 1D-H) and to only be dependent on the interconnected device upstream of the gene encoding the protein. The "default" network condition considered in this paper is illustrated in Figure 1A and 1D and its mathematical model of noise is herein discussed. A 3OC_6_-HSL-inducible input device is interconnected to a TetR-based NOT gate, whose output is measured; noise affects only OUTPUT_1_. Considering a vector of *N *inducer concentrations of 3OC_6_-HSL, at the i-th 3OC_6_-HSL concentration (i = 1...N), lognormal multiplicative noise (*v_i_*) is applied to the deterministic output (*y*_1*,i*_) of Module 1 (Eq.5-6).

(5) $$OUTPUT_{1,i}=y_{1,i}\cdot v_{i}$$

(6) $$v_{i}\sim LogN\left(0,\sigma_{i}^{2}\right)$$

where the logarithm of the lognormal distribution gives a Gaussian distribution with mean µ = 0 and variance $\sigma_{i}^{2}$, which, in general, depends on inducer concentration. The lognormal distribution is widely used to describe noise in biological processes and, in particular, it well describes the fluorescence distribution of reporter proteins in cell populations bearing synthetic gene networks, as it is often shown by experimental measurements performed via flow cytometry [45-48].

Under the hypotheses described above, the mean AVE and variance VAR of the lognormal noise are (Eq.7-8):

(7) $$AVE_{i}=e^{\frac{\sigma_{i}^{2}}{2}}$$

(8) $$VAR_{i}=e^{\sigma_{i}^{2}}\cdot \left(e^{\sigma_{i}^{2}}-1\right)$$

Since AVE is not 1, the average value of OUTPUT_1,i _is not *y*_1,*i*_, but it is (Eq.9):

(9) $$E\left[OUTPUT_{1,i}\right]=y_{1,i}\cdot AVE_{i}=y_{1,i}\cdot e^{\frac{\sigma_{i}^{2}}{2}}$$

which depends on $\sigma_{i}^{2}$. In the experiments performed in this paper, different noise entities are applied to Module 1; this would produce different average values for the same deterministic transfer function at a given induction (Eq.1). However, in this work we aim to study the effect of equal population-averaged inputs with different noise entities because instruments used in population-based experiments typically measure average values of reporter proteins. For this reason, we introduced a correction term: to have equal average values for OUTPUT_1 _for different noise variances, we corrected the deterministic output value *y*_1*,i *_as reported in Eq.10.

(10) $$y_{1,i}=y_{pop,i}\cdot e^{-\frac{\sigma_{i}^{2}}{2}}$$

where *y_pop,i _*is the population-averaged output of Module 1 at the i-th induction. From Eq.9 and Eq.10, the average output value for Module 1 is always (Eq.11):

(11) $$E\left[OUTPUT_{1,i}\right]=y_{pop,i}$$

Considering the Hill function of Module 1 (Eq.1), the *α*_*IN*,1 _and *δ*_*IN*,1 _parameters are scaled, with respect to their nominal values, as indicated in Eq.10, while the *k_IN _*and *η_IN _*parameters remain unchanged. This process enables to set identical population-averaged output values for different noise entities affecting the 3OC_6_-HSL-inducible module.

Two different noise models were considered: with constant coefficient of variation (CV) or with constant variance (VAR). The former has been experimentally observed in different works in the literature [13,32,33] and unpublished results from our laboratory, while the latter was used in this work to test a different assumption via simulations.

First, a constant CV noise model was assumed for all the 3OC_6_-HSL input concentrations. From Eq.7-8 it results that (Eq.12):

(12) $$CV=\frac{\sqrt{VAR_{i}}}{AVE_{i}}=\sqrt{e^{\sigma_{i}^{2}}-1}$$

Once a CV value is fixed, a value of σ^2 ^can be obtained (Eq.13):

(13) $$\sigma^{2}=\text{ln}\left(1+CV^{2}\right)$$

The σ^2 ^value computed from Eq.13 is independent from *y_pop,i _*and it is used to generate noisy samples for OUTPUT_1_.

Multiplicative lognormal noise model with constant VAR was also considered. From Eq.8 and Eq.10 it results that (Eq.14):

(14) $$Var\left[OUTPUT_{1,i}\right]=y_{pop,i}^{2}\cdot \left(e^{\sigma_{i}^{2}}-1\right)=VAR$$

From Eq.14, the $\sigma_{i}^{2}$ value can be obtained (Eq.15):

(15) $$\sigma_{i}^{2}=\text{ln}\left(1+\frac{VAR}{y_{pop,i}^{2}}\right)$$

In this case $\sigma_{i}^{2}$ is a function of *y_pop,i _*and is different for every i-th induction.

When noise was also applied to OUTPUT_2 _(see Figure 1E), multiplicative lognormal noise with constant CV or VAR was considered and generated by following the same concepts as above. In this case, noisy samples were extracted with a correlation coefficient 0<ρ<1 to model an extrinsic component of noise in addition to the intrinsic one. In particular, if noise is fully due to an intrinsic component, OUTPUT_1 _and OUTPUT_2 _are independent, since intrinsic noise is caused by the stochasticity in processes like gene expression. On the other hand, if part of the noise is due to an extrinsic component, OUTPUT_1 _and OUTPUT_2 _are correlated random variables, since biological processes like gene expression in each single cell are influenced by a pure stochastic component (intrinsic noise) and by fluctuations of cellular species (extrinsic noise) that are due to the variation between cells of resources like polymerases and ribosomes. In this context, the expression of two different genes shares common resources and, as a result, it will be correlated. The contribution of extrinsic noise is tuned by changing ρ between 0 (no extrinsic noise) and 1 (no intrinsic noise).

### Parameter values and implementation

The parameter values reported in [6] and obtained in unpublished experiments performed in our laboratory were used to describe the deterministic steady-state Hill functions of the nominal lux, tet and lac systems. Such values are reported for each simulation in the Results section. The CV values for multiplicative lognormal noise range between 15% and 75%, which are realistic values for cell-to-cell variability according to [26,31-33] and to unpublished experiments in our laboratory.

MATLAB R2010a (MathWorks, Natick, MA) was used to implement the study by generating data via *in silico *experiments and then performing parameter identification. For data generation, lognormal noise with specific entity was applied to the Hill function outputs, which were propagated throughout the cascade of interconnected modules in the network. The Hill functions were computed as described in Eq.1-4, while the lognormal noise was multiplied to the outputs according to Eq.5-6 and Eq.10. The *lognrnd *function was used to generate independent lognormal noise samples. Correlated lognormal noise samples were computed as *exp(vn)*, where *vn *are Gaussian noise samples obtained via the *mvnrnd *function. For each *in silico *experiment at the i-th 3OC_6_-HSL concentration, 10,000 independent lognormal noise samples were extracted and used to generate synthetic data, which simulate the steady-state output of 10,000 sampled cells. The *lsqnonlin *routine was used for parameter estimation. Sensitivity analyses were performed by changing parameters individually in the value ranges specified in the Results section, while all the other parameters were fixed at their nominal values. Parameters were changed by spanning a range of plausible values, according to a number of published [6,19,42,49] and unpublished *in vivo *experiments carried out by our group. The aim of sensitivity analyses was to evaluate the impact of parameter values on the variability of the parameter estimates.

## Results and discussion

### Transfer function identification for a single module

#### Characterization of a TetR/Ptet-based NOT gate

**Figure 2:**
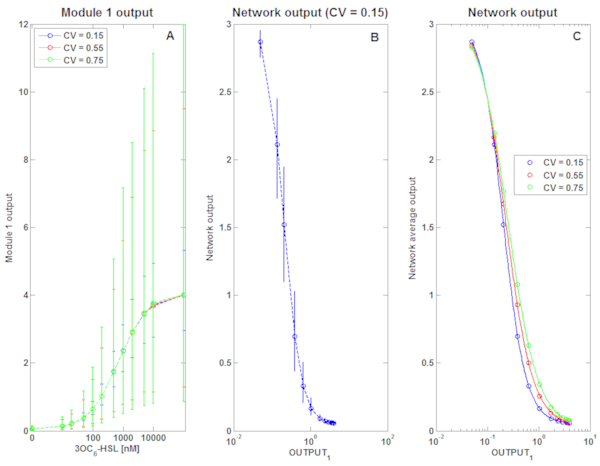
**OUTPUT_1 _and OUTPUT_2 _signals in the two-module network (including the TetR/Ptet-based NOT gate) with constant CV noise model**. A) OUTPUT_1 _signal for different noise entities, in response to 3OC_6_-HSL; in all the graphs, data points represent population-averaged values and error bars represent 95% confidence intervals. B) OUTPUT_2 _signal as a function of average OUTPUT_1 _in case of CV = 0.15. The average OUTPUT_1 _is computed from the 3OC_6_-HSL concentrations from panel A. Data points and error bars have the same meaning as above. Here, the cell-to-cell variability is derived from the propagation of noise from OUTPUT_1_. C) Population-averaged OUTPUT_2 _values as a function of average OUTPUT_1_. For all the CV values, data are fitted with a Hill function (solid line). The estimated parameters are reported in Table 2.

The two-module gene network illustrated in Figure 1A was considered as model system and it was analyzed in its default conditions: OUTPUT_1 _was affected by multiplicative lognormal noise with constant CV, while OUTPUT_2 _was assumed to be unaffected by noise (see Figure 1D). Hill functions with parameters reported in Table 1 were used to simulate the behaviour of the two modules [6]. CV values were set to 0.15, 0.55 and 0.75. For each CV value, population-averaged measures of OUTPUT_1 _are shown, with their 95% confidence intervals, as a function of 3OC_6_-HSL concentration (Figure 2A); then, population-averaged values of OUTPUT_2 _are reported in a representative condition (for clarity of presentation, only the graph relative to one CV value is reported, see Figure 2B) with their 95% confidence intervals derived from the propagation of noise from the upstream module; finally, the population-averaged values are shown and the corresponding fitted curves are also reported (Figure 2C). By fitting the population-averaged OUTPUT_2 _against OUTPUT_1 _for each CV, the parameters of the NOT module were estimated and their values are reported in Table 2. Given a noise model and entity, the CV was computed for the *α_OUT_, k_OUT _*and *η_OUT _*parameters. Since in all cases the δ parameter was very low and difficult to accurately estimate [6], it exhibited a very large CV; for this reason, only the variability of the other parameters will be discussed.

**Table 1:** Parameter sets used to describe the steady-state transfer function of the genetic devices

Parameter	*α*	*δ*	*k*	*η*
**3OC_6_-HSL inducible device**	4	0.05	700	0.9

**TetR/Ptet NOT gate**	3	0.05	0.2	2

**LacI/Plac NOT gate**	0.5	10^-5^	3.2	1.9

**A/PA YES gate**	3	0.05	0.2	2

The α, δ, K and η parameters correspond to the α_IN_, δ_IN_, k_IN _and η_IN _parameters of the 3OC_6_-HSL inducible device (Eq.1), α_OUT_, δ_OUT_, k_OUT _and η_OUT _of the TetR/Ptet-based NOT gate and of the A/PA-based YES gate (Eq.2 and Eq.4) and α_OUT3_, δ_OUT3_, k_OUT3 _and η_OUT3 _of the LacI/Plac-based NOT gate (Eq.3). Their units are described in the Methods section.

**Table 2:** Estimated parameters for Module 2 as a function of noise model and entity.

Parameter:	*α_OUT_* [RPU]	*δ_OUT_* [RPU]	*k_OUT_* [RPU]	*η_OUT_* [-]
**TetR/Ptet (constant CV)**	3.00 3.04 3.06 (1%)	0.05 0.04 0.02	0.20 0.23 0.25 (10.5%)	1.96 1.65 1.49 (14.2%)

**TetR/Ptet (constant VAR)**	2.85 2.87 2.87 (0.4%)	0.04 0.04 0.03	0.27 0.30 0.32 (9.4%)	2.53 2.18 2.18 (0.6%)

**LacI/Plac (constant CV)**	0.50 0.46 0.43 (6.6%)	0 0.04 0.07	3.20 3.27 3.32 (1.8%)	1.88 1.70 1.60 (8.2%)

**LacI-Plac (constant VAR)**	0.50 0.50 0.50 (0.2%)	0 0 0	3.22 3.23 3.25 (0.4%)	1.90 1.90 1.89 (0.2%)

**A-PA (constant CV)**	3.00 3.04 3.06 (1%)	0.05 0.02 0.02	0.20 0.23 0.25 (10.5%)	1.96 1.65 1.49 (14.2%)

**A-PA (constant VAR)**	2.85 2.87 2.87 (0.4%)	0.21 0.19 0.19	0.27 0.30 0.32 (9.4%)	2.15 2.18 2.18 (0.6%)

Parameters are obtained by fitting population-averaged values of OUTPUT_2 _as a function of OUTPUT_1 _for different noise models and entity, applied to OUTPUT_1_. The three values reported in each cell correspond to CV = 0.15, 0.55, 0.75 (for constant CV models) and to VAR = 0.05, 0.1, 0.15 (for constant VAR models). The CV among the estimated parameters is reported in brackets.

First, the TetR/Ptet-based NOT gate will be considered and the results obtained by simulating this system are reported. The variability of parameters was very low, with *η_OUT _*exhibiting the largest variation (CV of 14.2%). A CV of 9.4% (for *k_OUT_*) was obtained when the same gene network was tested assuming a lognormal noise model with constant variance, set to 0.05, 0.1 and 0.15, which provides different cell-to-cell variability entities, although not directly comparable to the constant CV model variability levels (see Additional file 1: Figure S1). This variability value is again very low. The results obtained demonstrate that, in the tested network, population-averaged data can be used to measure the input-output transfer function of the NOT gate with a low variability, even if input devices affected by significantly different cell-to-cell variability are used. However, it is important to highlight that the measured parameters are not identical to the ones used to generate the data (reported in Table 1), herein called "true" parameters. The *η_OUT _*parameter exhibited the highest maximum percentage difference (25.7%) for lognormal noise with constant CV and represents a relatively low difference; on the other hand, the highest maximum percentage difference observed for lognormal noise with constant VAR was for *k_OUT _*(61.8%, see Table 2 and Additional file 1: Figure S1) and represents a higher difference entity. This high variability in the estimated *k_OUT _*parameter might be due to the high cell-to-cell variability of the 3OC_6_-HSL-inducible module around the true *k_OUT _*value of the NOT gate (0.2 RPU, see Additional file 1: Figure S1A), as opposed to the variability shown in Figure 2A. This variability propagates and causes higher cell-to-cell variability in OUTPUT_2 _when OUTPUT_1 _is close to *k_OUT _*(compare Figure 2B and Additional file 1: Figure S1B). The described phenomenon is marked because the NOT gate is very sensitive to Module 1 output variation, that is, input values similar to the *k_OUT _*parameter are reached at very low 3OC_6_-HSL concentrations. This situation is sometimes desired to create switches with steep responses [32], but when the input device is affected by large-entity noise at low-induction levels the NOT gate could propagate and amplify this noise and the transfer function parameters of the NOT gate, estimated from central tendency measures, would also be affected. The measurement of the steady-state transfer function of highly-sensitive switches with tuneable upstream modules also results in the inability to span their full activity range, that is, OUTPUT_2 _could never reach its maximum value *δ_OUT _*+ *α_OUT _*even for very low OUTPUT_1 _values. In the configuration presented in Figure 2B,C the activity range of the NOT gate is almost fully covered by OUTPUT_1_, but for low OUTPUT_1 _values the activity is already decreasing; this effect can make the estimation of transfer function parameters difficult in real experiments, where the number of data points might be limited. The described effects are widely known and drive the choice of suitable input devices whose activity range matches the activity range of the device of interest they are supposed to drive [6,50,51].

#### Characterization of a LacI/Plac-based NOT gate

We performed the same analysis described above considering a NOT gate with much lower sensitivity (a LacI/Plac-based NOT gate was used as Module 2, whose transfer function parameters are shown in Table 1) to the input module. The variability entity of estimated parameters is very low and the maximum percentage difference with true parameters is significantly lower (see Table 2 for estimated parameters) than for the TetR/Ptet-based NOT gate, yielding a CV of 15.7% (*η_OUT_*) and 1.5% (*k_OUT_*) in the constant CV and VAR conditions, respectively. The fitted curves are reported in Additional file 1: Figure S2 and Figure S3. Even if this difference is lower, this circuit highlights another widely known limit in matching input-output activity ranges; in fact, the lower part of the NOT gate transfer function is not observable, since OUTPUT_1 _cannot reach the required maximum activity (see Additional file 1: Figure S2C and Figure S3C). As explained above, this effect can complicate the identification of transfer functions in real experiments.

#### Characterization of a YES gate

When the NOT gate is replaced by a YES gate, that is, a device with an opposite logic behaviour, with the same Hill function parameters as the TetR/Ptet-based NOT gate (see Table 1), results do not change significantly when compared to the NOT gate: *η_OUT _*is the most variable (CV of 14.2%) in the constant CV model condition, while *k_OUT _*has the highest CV (9.3%) in the constant VAR model. In both cases variability is very low. The maximum percentage difference between estimated and true parameters is also limited, with maximum values of 25% (*η_OUT_*, constant CV model) and 61% (*k_OUT_*, constant VAR model). The fitted curves are shown in Additional file 1: Figure S4 and Figure S5. From Additional file 1: Figure S4B and Figure S5B, it is important to highlight that, since the switch is very sensitive to the input device, the lower part of the curve is not fully observable and this results in *δ_OUT _*estimates with low accuracy. This problem is analogous to the one observed for the TetR/Ptet-based NOT gate (see above).

#### Sensitivity analysis

**Figure 3:**
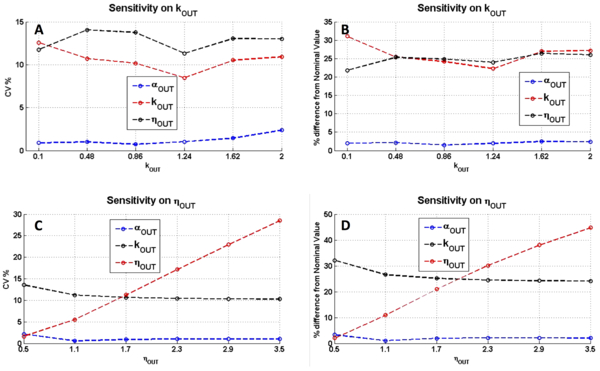
**Sensitivity analysis for the two-module network with the TetR/Ptet-based NOT gate, when OUTPUT_1 _is affected by constant CV noise: variability among the estimated parameters and maximum percentage difference between estimated and true parameters**. CV among the estimated parameters (A,C), and maximum percentage difference between estimated and true parameters (B,D) for different values of *k_OUT _*(A-B) and *η_OUT _*(C-D). CV was computed among the parameters estimated for noise CV = 0.15, 0.55 and 0.75.

The conditions tested so far depicted different effects whose entity depends on the specific network considered, although results do not change by switching the logic of the module of interest from repression to activation. Because it is hard to define general rules to analyze the effects of noise in population-averaged input-output measurements, we performed a sensitivity analysis on the two-module network with the TetR/Ptet-based NOT gate to explore the system response for different parameter configurations and to estimate the parameters of highest impact. For each noise model and entity, two parameters were tested: *η_OUT _*was varied from 0.5 to 3.5 and *k_OUT _*from 0.1 to 2. Results for the constant CV noise model are shown in Figure 3 in terms of CV among estimated parameters and of maximum percentage difference with true value. The variability among the estimated parameters appears not to be significantly correlated with *k_OUT _*variations for all the tested values; in particular, the estimated *k_OUT _*and *η_OUT _*exhibit a CV between 8% and 15%, while *α_OUT _*has a CV lower than 2.5% (see Figure 3A). The maximum percentage difference of estimated parameters shows the same trend as a function of *k_OUT_*, with *k_OUT _*and *η_OUT _*exhibiting a difference of 20-30%, while *α_OUT _*of less than 2.5% (see Figure 3B). When considering the variability of estimated parameters as a function of *η_OUT_*, the CV among *k_OUT _*and *α_OUT _*appears to be uncorrelated as above, with a CV below 15% and 2.5%, respectively; conversely, *η_OUT _*exhibits a linear increase of CV, up to 28% for the tested values of varied *η_OUT _*(see Figure 3C). The maximum percentage difference between estimated parameters and true value shows the same trend as above as a function of varied *η_OUT_*, with a linear trend of *η_OUT _*reaching 45% for the tested values (see Figure 3D).

When considering constant VAR noise model, trends are different (see Additional file 1: Figure S6). By varying *k_OUT _*values, the CV among estimated parameters is relatively constant for *η_OUT _*and *α_OUT _*(below 2%), while the one of *k_OUT _*shows a decreasing trend, which is of relatively low entity, below 12% (see Additional file 1: Figure S6A). The maximum percentage difference shows a similar trend, with *k_OUT _*exhibiting a difference up to 120%, with the other parameters showing a difference below 20% (see Additional file 1: Figure S6B). On the other hand, by varying *η_OUT_*, the CV among estimated parameters is always relatively low (below 17%); in particular, *k_OUT _*decreases from 17% (for *η_OUT _*= 0.5) to about 10%, *α_OUT _*decreases from 4% (for *η_OUT _*= 0.5) to a value below 1%, while *η_OUT _*shows a U-like trend with a minimum in *η_OUT _*= 2.3 (see Additional file 1: Figure S6C). The maximum percentage difference between estimated and true parameters also depicts the same U-like trend for *η_OUT _*whose difference varies from 55% to less than 2% (see Additional file 1: Figure S6D); as for the other parameters, the difference in *k_OUT _*is around 60% for all the tested *η_OUT _*values except for *η_OUT _*= 0.5 where the difference is 30%; finally, *α_OUT _*shows a minor difference (below 10%).

#### Contribution of extrinsic noise

**Figure 4:**
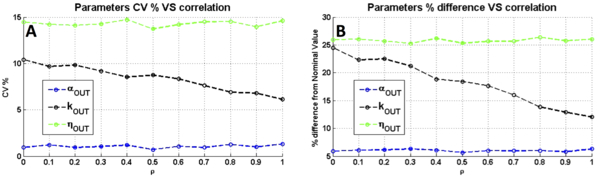
**Analysis of the two-module network with the TetR/Ptet-based NOT gate, when OUTPUT_1 _and OUTPUT_2 _are affected by constant CV noise with correlation coefficient ρ**. A) Variability among the estimated parameters, in terms of CV. B) Maximum percentage difference between estimated and true parameter values. All the results are shown as a function of the correlation coefficient ρ, which is varied from 0 (no correlation) to 1 (maximum correlation). The increase of ρ value simulates an increase in proportion of the extrinsic component of noise over the total noise, which is composed of the intrinsic and extrinsic components.

In a realistic framework, noise would also affect OUTPUT_2_. To test this condition, lognormal noise with constant CV was applied to OUTPUT_1 _(CV of 15%, 55% and 75%) and OUTPUT_2 _(CV of 15%) with a correlation coefficient ρ, in the two-module network with the TetR/Ptet-based NOT gate. The results in terms of estimated parameters variability among the OUTPUT_1 _noise entities are reported in Figure 4A. As it was performed above, the maximum percentage difference between the estimated parameters and the true ones was also computed and reported as a function of ρ (see Figure 4B). For comparisons, the true *α_OUT _*and *δ*_*OUT *_parameters were rescaled as indicated in Eq.10 to consider the average value of multiplicative lognormal noise which is different from 1 (see Methods section for details). Results depict that the variability and percentage difference in *η_OUT _*and *α_OUT _*are not affected by ρ, showing a constant CV of about 15% for *η_OUT _*and 1.5% for *α_OUT _*(see Figure 4A) and a maximum percentage difference of about 25% for *η_OUT _*and 6% for *α_OUT _*(see Figure 4B). On the other hand, considering *k_OUT_*, CV and maximum percentage difference are affected by ρ, both showing a 2-fold decrease from ρ = 0 to ρ = 1. In particular, a linear decrease of CV from 10% to 6% (see Figure 4A) and a decrease of percentage difference from 25% to 12% (see Figure 4B) are observed.

By assuming a constant VAR model for OUTPUT_1 _(VAR values of 0.05, 0.1 and 0.15) and OUTPUT_2_, (VAR value of 0.15), variability and percentage difference trends are different from the constant CV case (see Additional file 1: Figure S7). In this case, CV does not show any strong trend as a function of ρ, except for *k_OUT _*which shows a variability decrease from 10% to 6% (see Additional file 1: Figure S7A); in contrast, the maximum percentage difference between true and estimated *η_OUT _*parameter shows a 3-fold linear increase, from 10% (for ρ = 0) to 30% (for ρ = 1); *k_OUT _*shows a decreasing trend of high entity, from ~62% (for ρ = 0) to 30% (for ρ = 1); finally, *α_OUT _*does not exhibit specific trends and shows a difference value below 10%.

The same process was performed by considering the YES gate instead of the TetR/Ptet-based NOT gate and the results, shown in Additional file 1: Figure S8, do not quantitatively change for constant CV noise model (see Additional file 1: Figure S8A, B), but for constant VAR noise the maximum percentage difference of all the parameters increases as a function of ρ, while the CV among the estimated parameters shows a low variability, up to 10% (see Additional file 1: Figure S8C, D).

Overall, the presence of an extrinsic component in the total noise can improve the accuracy in the estimation of some transfer function parameters, by decreasing the variability of the *k_OUT _*parameter in different noise model and entity conditions, and by decreasing the maximum percentage difference between estimated and true *k_OUT _*parameter for the NOT gate; however, the maximum percentage difference of the *η_OUT _*parameter for the NOT gate increases for increasing values of ρ and, in the case of the YES gate with constant VAR noise, the CV of *η_OUT _*and the maximum percentage difference of all the three parameters increases with ρ. These results demonstrate that changing the logic of the module of interest can affect the overall results when correlated noise is assumed. A possible explanation of these results is that a correlated noise model propagates the variability of OUTPUT_1 _in a different way as a function of Module 2 logic. In fact, the cell-to-cell variability observed for ρ = 0 decreases for ρ = 0.5 (a realistic proportion of extrinsic noise component [26]) when considering the NOT gate with constant VAR model (see Additional file 1: Figure S9A and Figure S9B), while it increases when considering the YES gate (see Additional file 1: Figure S9C and Figure S9D). The same trend is also observed for constant CV noise (see Additional file 1: Figure S10A-D). This effect is probably due to the amplification of noise by the YES gate for increasing correlation values, since ρ>0 and Module 2 has an increasing activity as a function of OUTPUT_1_; on the other hand, the NOT gate decreases the inter-individual variability, since it has a decreasing activity as a function of OUTPUT_1_, thus compensating for the noise applied upstream.

### Input-output function identification for an interconnected network

In contrast to the previous section, where the aim was to identify the transfer function of a single module, in this case the input-output function of a three-module interconnected network (see Figure 1C) is identified as a black-box, whose behaviour is described by the Hill equation (Eq.16):

(16) $$OUTPUT_{3}=\delta_{OUT}^{*}+\frac{\alpha_{OUT}^{*}}{1+{\left(\frac{k_{OUT}^{*}}{3OC_{6}-HSL}\right)}^{\eta_{OUT}^{*}}}$$

where 3OC_6_-HSL is the input, OUTPUT_3 _is the output of the whole network and parameters $\alpha_{OUT}^{*}$, $\delta_{OUT}^{*}$, $k_{OUT}^{*}$ and $\eta_{OUT}^{*}$ have the same meaning as in the Methods section.

#### Three-module network prediction from individually-characterized modules

We performed simulated experiments where: i) the transfer function of each single module was identified from population-averaged values (as performed in the previous section) and ii) the identified transfer functions were used to predict the black-box input-output function of the interconnected network. This process was repeated for each noise model and entity considered and Hill equation parameters were estimated for the black-box transfer function.

Considering the two-module networks with TetR/Ptet- and LacI/Plac-based NOT gates (see Figure 1A), the parameters reported in Table 1 were used to generate data, assuming the constant CV and VAR noise models, only applied to OUTPUT_1 _(see Figure 1D). From an experimental point of view, the described procedure aims to simulate the transfer function learning for individual modules via central tendency measures (on two-module networks), and the prediction of the population-averaged output of a complex function (a three-module network) built up by interconnecting such modules.

The parameters describing the transfer function of individual modules were obtained previously (see Table 2), while the estimated black-box function parameters are reported in Table 3 with their CV. In practice, the TetR/Ptet- and the LacI/Plac-based NOT gate transfer functions were identified assuming the noise model and entity reported in Table 2 thus obtaining different parameter estimates; then, the black-box transfer function of the three-module network was predicted by using the TetR/Ptet- and the LacI/Plac-based NOT gate transfer functions with these parameter sets, thus obtaining 9 transfer function combinations for each noise model (see Table 3).

**Table 3:** Estimated parameters for the three-module network considered as a black-box function, for different noise models and entities, when the function is predicted from individual transfer functions derived from central tendency measures

Parameter:	$\alpha_{OUT}^{*}$ [RPU]	$\delta_{OUT}^{*}$ [RPU]	$k_{OUT}^{*}$ [nM]	$\eta_{OUT}^{*}$ [-]
**constant CV**	0.22 0.20 0.19 0.22 0.20 0.19 0.22 0.20 0.19 (6.5%)	0.28 0.30 0.31 0.28 0.30 0.31 0.28 0.30 0.31	16.86 18.51 19.53 20.00 22.25 23.67 23.03 25.84 27.63 (16%)	1.75 1.69 1.65 1.53 1.48 1.45 1.42 1.37 1.34 (9.7%)

**constant VAR**	0.22 0.22 0.21 0.22 0.22 0.22 0.22 0.22 0.22 (0.8%)	0.28 0.28 0.28 0.28 0.28 0.28 0.28 0.28 0.28	23.98 23.96 23.97 27.73 27.70 27.72 31.22 31.19 31.21 (11.3%)	1.90 1.90 1.89 1.92 1.92 1.92 1.92 1.92 1.92 (0.7%)

Parameters are obtained by fitting population-averaged values of OUTPUT_2 _as a function of OUTPUT_1 _for different noise models and entity, applied to OUTPUT_1_. The 9 values reported in each cell correspond to a noise entity (which affects OUTPUT_1 _in the identification step of the TetR/Ptet- and LacI/Plac-based NOT gate transfer function, respectively) of CV = 0.15-0.15, 0.15-0.55, 0.15-0.75, 0.55-0.15, 0.55-0.55, 0.55-0.75, 0.75-0.15, 0.75-0.55, 0.75-0.75 (for constant CV models) and to VAR = 0.05-0.05, 0.05-0.1, 0.05-0.15, 0.1-0.05, 0.1-0.1, 0.1-0.15, 0.15-0.05, 0.15-0.1, 0.15-0.15 (for constant VAR models). The CV among the estimated parameters is reported in brackets.

These results depict that the resulting variability is very low, with the highest CV value for $k_{OUT}^{*}$, 16% and 11.3%, in the constant CV noise model and in the constant VAR model, respectively.

#### Comparison between network predictions from individually-characterized modules and deterministic output of the circuit

As already discussed in the case of the characterization of a single module, the parameters obtained in the previous study can be different from the ones estimated in a deterministic framework (reported in Table 4), that is, without noise. For this reason, their maximum percentage difference was computed. In both the constant CV and VAR noise models, the $k_{OUT}^{*}$ parameter is affected by the highest difference (68.3% and 90.2%, respectively), thus showing a moderately high deviation. Conversely, the other parameters give a maximum difference of 24.8% and 8.3%, both on $\eta_{OUT}^{*}$, in the constant CV and VAR case, respectively. This deviation indicates that, in the investigated system with the specific parameters and noise assumed, the contribution of noise is significant; for this reason, given the knowledge of the real transfer functions of the modules, the input-output function identification of the whole network is affected by a maximum error of 68.3% (constant CV) or 90.2% (constant VAR) if noise is not considered.

**Table 4:** Estimated parameters for the three-module network considered as a black-box function, without noise affecting the **network**.


$\alpha_{OUT}^{*}$ [RPU]	$\delta_{OUT}^{*}$ [RPU]	$k_{OUT}^{*}$ [nM]	$\eta_{OUT}^{*}$ [-]

0.22	0.28	16.42	1.78

Parameters are obtained by fitting deterministic values of OUTPUT_3 _as a function of 3OC_6_-HSL.

#### Comparison between network predictions from individually-characterized modules and network output generated in the presence of noise

To extend the study on the three-module network, another simulated experimental study was performed. The parameters of the black-box function predicted from central tendency measures (reported in Table 3) were compared to the parameters of the black box function simulated by using the network of Figure 1H (generated by using the parameters of Table 1). In the latter case, as indicated in Figure 1H, noise was applied to both OUTPUT_1 _and OUTPUT_2_, and the $\alpha_{OUT}^{*}$, $\delta_{OUT}^{*}$, $k_{OUT}^{*}$ and $\eta_{OUT}^{*}$ parameters were estimated from population-averaged OUTPUT_3 _measures (see Table 5); only constant CV noise was considered and applied with different entities (CV of 0.15, 0.55 and 0.75). In practice, the 9 parameter sets combinations reported in Table 3 were compared to the 9 parameter sets combinations reported in Table 5. The identification results show that very low variability occurs among the estimated parameters (see CV in Table 5). When comparing the 9 parameter sets of Table 3 to the 9 sets of Table 5 (thus performing 81 comparisons), the maximum percentage difference was 65.9% (for the $k_{OUT}^{*}$ parameter) that was observed in the comparison between the condition where noise with a CV of 0.15 affects OUTPUT_1 _in the identification step of both the TetR/Ptet- and the LacI/Plac-based NOT gates (see Table 3), and the network condition in which OUTPUT_1 _and OUTPUT_2 _are both characterized by a noise with a CV of 0.75, which propagates towards OUTPUT_3 _(see Table 5). This result indicates that if the transfer function of individual modules is identified via central tendency measures data when noise is low (CV of 15%) and these learnt functions are used to predict the output of the three-module network, the outputs have a maximum difference of 65.9% (estimated on the $k_{OUT}^{*}$ parameter) if the network is affected by a noise of larger entity on both OUTPUT_1 _and OUTPUT_2_. This can be considered as a low-entity difference (less than 2-fold) when compared to the possible large prediction errors performed when pre-characterized modules are interconnected and tested [6,15,18,39].

**Table 5:** Estimated parameters for the three-module network considered as a black-box function, for different noise entities and constant CV noise model, when the function is simulated by using the three-module network of Figure 1H.

Parameter:	*α_OUT_* [RPU]	*δ_OUT_* [RPU]	*k_OUT_* [RPU]	*η_OUT_* [-]
**constant CV**	0.22 0.22 0.22 0.20 0.20 0.20 0.19 0.19 0.19 (6.5%)	0.28 0.28 0.28 0.30 0.30 0.30 0.31 0.31 0.31	16.90 20.78 24.42 18.90 23.10 25.91 19.32 23.71 27.97 (16.2%)	1.73 1.41 1.26 1.73 1.41 1.22 1.66 1.38 1.24 (14.3%)

Parameters are obtained by fitting population-averaged values of OUTPUT_2 _as a function of OUTPUT_1 _for different noise models and entity, applied to OUTPUT_1_. The 9 values reported in each cell correspond to a noise (which affects OUTPUT_1 _and OUTPUT_2_, respectively) of CV = 0.15-0.15, 0.15-0.55, 0.15-0.75, 0.55-0.15, 0.55-0.55, 0.55-0.75, 0.75-0.15, 0.75-0.55, 0.75-0.75. The CV among the estimated parameters is reported in brackets.

Supplementary results are reported in Additional file 1 where a two-module network including the TetR/Ptet-based NOT gate (see Figure 1A) is also studied via an analogous procedure and a sensitivity analysis is performed on its structural parameters.

## Conclusions

In this work we have evaluated the contribution of noise in two different situations, via simulated *in silico *studies.

First, we have tested the identification of an individual module (a NOT gate) via an interconnected network composed of two modules. The results highlighted that central tendency measures can be used accurately to summarize the transfer function of the single module, since the estimated parameters are affected by a low CV (up to 14.2%). However, a larger percentage deviation (up to 61.8%) is observed when comparing the estimated parameters with the true ones, which generated the data. For these reasons, the expected differences (caused by noise) in transfer function identification when using different input devices upstream are low, while an accurate measurement of the true transfer function requires, in addition, the full knowledge of noise.

All these results have been found to be dependent on the structural parameters of the module of interest and, for this reason, a sensitivity analysis was carried out to elucidate the CV and maximum percentage difference trends as a function of such parameters. On the other hand, the logic of the module of interest was not important in this step, since the replacement of a NOT gate with a YES gate with the same structural parameters does not change the conclusions. Model refinement, including an extrinsic component of noise, also elucidated a trend in CV and maximum percentage difference, which, in this case, are logic-dependent.

For the above reasons, in the considered case studies noise should be included in models if the aim is to estimate the real parameter values of individual transfer functions. This requires the knowledge of noise model, which can be inferred, for example, by flow cytometric analyses. However, if the aim is to characterize and re-use a biological module, noise can be omitted since different noise entities (generated by the input module used for the characterization purpose) do not provide significant changes in estimated parameters. As a result, in the tested conditions, the noise of an input module is not responsible for changes in the estimated parameters of the module of interest.

Second, we have tested the identification of a black-box input-output function predicted by interconnecting the three genetic modules that were previously characterized *in silico*, in the presence of noise, via central tendency-based measurements to identify their transfer function. Noise entity did not significantly affect the input-output function identification. However, as observed before for the single module identification, noise significantly affects the percentage difference between estimated input-output function and the function obtained in absence of noise (up to 90.2%).

As a last study involving the black-box input-output transfer function of a three-module network, we compared the black-box function predicted by using the individual modules identified from data generated with different noise entities affecting OUTPUT_1_, and the black-box function simulated by using different noise entities affecting OUTPUT_1 _and OUTPUT_2_. A maximum percentage difference of 65.9% was observed. This has high relevance in the bottom-up design of gene networks with predictable function. In fact, this difference represents the maximum variability, considering noise as the sole variability source, that can be observed between i) the prediction of the black-box function of a network from the knowledge of individual modules and ii) a black-box function that is generated by the same modules, when the noise affecting the identification step is different from the noise in the final circuit context. The changing of cell-to-cell variability entity when the context is different is a common situation in biological engineering, in which characterized modules are re-used in different contexts to engineer complex interconnected networks.

The same results were confirmed on a two-module network, on which a sensitivity analysis is also reported (see Additional file 1: Supplementary results).

As anticipated above, all the obtained results were dependent on the specific network parameters used; for this reason, the conclusions obtained in this work are confined to the topology and parameter ranges here assumed, although the work can be easily extended to study different systems, according to biological engineers' needs.

On the other hand, in the working context described above, the relevance of this study is high in biological engineering, since it can effectively guide the experimental work of systems and synthetic biologists to carry out suitable *in vivo *measurements (population/central tendency-based approaches or single-cell ones) and it can be a crucial tool to accurately distinguish actual non-modular and unpredictable phenomena from the effects due to noise in the interconnection of biological parts to construct complex gene networks from the bottom-up. As a practical example, in one of our experimental studies [6] we found a CV of 44% (for the *k_OUT _*parameter) when the transfer function of a TetR/Ptet-based NOT gate was identified from population-based measurements, performed via different input devices assembled upstream. The NOT gates drove the expression of a reporter gene to easily visualize the output. Experimental measurements consisted in population-based fluorescent protein quantification in recombinant cultures. A microplate reader was used to incubate cultures and perform absorbance and fluorescence measurements. Considering the exponential growth phase of bacterial cultures, absorbance and fluorescence time series were processed to obtain an individual value representing the activity of the NOT gate device, expressed as Relative Promoter Units (RPUs), in response to different input devices and induction levels. Although the cell-to-cell variability was not experimentally measured for the input devices outputs, we can conclude that the CV among the estimated *k_OUT _*parameters of the NOT gate could not be caused by the sole noise (which gives contributions up to ~14%), thus highlighting a non-modular behaviour of the used components, although, in principle, part of the total variability could be caused by heterogeneity of cells. By refining the study with the characterization of the cell-to-cell variability of each individual module, the knowledge of the true NOT gate parameters can be obtained, since the ones estimated in the presence of noise can deviate from the true ones (according to this study, by a maximum percentage difference of ~62%). As gene networks with the architecture studied in this work are widely used in synthetic biological circuits, the proposed approach can be useful to support the characterization and re-use of modules in different circuits and to support the prediction of interconnected circuit output.

It is worth noting that cell-to-cell variability is a very complex element, which (given a gene network) may be dependent on the specific strain and environmental context [3], dynamics of gene expression and specific biological processes [29]. Finally, cell-to-cell variability may not be solely explained by intrinsic and extrinsic noise, but it might depend on bistability effects in gene regulatory networks [52] or on the evolutionary context that has been found to significantly affect the quantitative behaviour of single cells in several experimental studies, by increasing the failure rate of a biological function [3,44,49,53,54].

## Competing interests

The authors declare that they have no competing interests.

## Authors' contributions

NP, LP and PM designed the study. NP performed the study. NP, LP, SZ and PM analyzed the data. NP, LP and PM wrote the paper.

## Supplementary Material

Additional file 1 **Supplementary figures, results and tables**. Click here for file

## Declarations

Publication of this article has been funded by the University of Pavia.

This article has been published as part of *BMC Systems Biology *Volume 9 Supplement 3, 2015: Proceedings of the Italian Society of Bioinformatics (BITS): Annual Meeting 2014: Systems Biology. The full contents of the supplement are available online at http://www.biomedcentral.com/bmcsystbiol/supplements/9/S3.

## References

[B1] Endy D Foundations for engineering biology Nature 2005 438 7067 449 453 10.1038/nature04342 16306983

[B2] Lu TK Khalil AS Collins JJ Next-generation synthetic gene networks Nat Biotechnol 2009 27 12 1139 1150 10.1038/nbt.1591 20010597 PMC2796205

[B3] Arkin AP A wise consistency: engineering biology for conformity, reliability, predictability Curr Opin Chem Biol 2013 17 6 893 901 10.1016/j.cbpa.2013.09.012 24268562

[B4] Sprinzak D Elowitz MB Reconstruction of genetic circuits Nature 2005 438 7067 443 448 10.1038/nature04335 16306982

[B5] Kwok R Five hard truths for synthetic biology Nature 2010 463 7279 288 290 10.1038/463288a 20090726

[B6] Pasotti L Politi N Zucca S Cusella De Angelis MG Magni P Bottom-up engineering of biological systems through standard bricks: a modularity study on basic parts and devices PLoS One 2012 7 7 e39407 10.1371/journal.pone.0039407 22911685 PMC3401228

[B7] Pasotti L Zucca S Advances and computational tools towards predictable design in biological engineering Comput Math Methods Med 2014 2014 369681 25161694 10.1155/2014/369681 PMC4137594

[B8] Del Vecchio D Ninfa AJ Sontag ED Modular cell biology: retroactivity and insulation Mol Syst Biol 2008 4 161 18277378 10.1038/msb4100204 PMC2267736

[B9] Sauro HM Modularity defined Mol Syst Biol 2008 4 166 18277382 10.1038/msb.2008.3 PMC2267732

[B10] Zhang F Carothers JM Keasling JD Design of a dynamic sensor-regulator system for production of chemicals and fuels derived from fatty acids Nat Biotechnol 2012 30 4 354 359 10.1038/nbt.2149 22446695

[B11] Paddon CJ Keasling JD Semi-synthetic artemisinin: a model for the use of synthetic biology in pharmaceutical development Nat Rev Microbiol 2014 12 5 355 367 10.1038/nrmicro3240 24686413

[B12] Cameron DE Bashor CJ Collins JJ A brief history of synthetic biology Nat Rev Microbiol 2014 12 5 381 390 10.1038/nrmicro3239 24686414

[B13] Guido NJ Wang X Adalsteinsson D McMillen D Hasty J Cantor CR A bottom-up approach to gene regulation Nature 2006 439 7078 856 860 10.1038/nature04473 16482159

[B14] Hajimorad M Gray PR Keasling JD A framework and model system to investigate linear system behavior in Escherichia coli J Biol Eng 2011 5 3 10.1186/1754-1611-5-3 21510907 PMC3110104

[B15] Davis JH Rubin AJ Sauer RT Design, construction and characterization of a set of insulated bacterial promoters Nucleic Acids Res 2011 39 3 1131 1141 10.1093/nar/gkq810 20843779 PMC3035448

[B16] Wang B Kitney RI Joly N Buck M Engineering modular and orthogonal genetic logic gates for robust digital-like synthetic biology Nat Commun 2011 2 508 22009040 10.1038/ncomms1516 PMC3207208

[B17] Moon TS Lou C Tamsir A Stanton BC Voigt CA Genetic programs constructed from layered logic gates in single cells Nature 2012 491 7423 249 253 10.1038/nature11516 23041931 PMC3904217

[B18] Ceroni F Furini S Stefan A Hochkoeppler A Giordano E A synthetic post-transcriptional controller to explore the modular design of gene circuits ACS Synth Biol 2012 1 5 163 171 10.1021/sb200021s 23651154

[B19] Zucca S Pasotti L Mazzini G De Angelis MGC Magni P Characterization of an inducible promoter in different DNA copy number conditions BMC Bioinformatics 2012 13 Suppl 4 S11 10.1186/1471-2105-13-S4-S11 22536957 PMC3314568

[B20] Pasotti L Zucca S Magni P Modelling for Synthetic Biology Modeling Methodology for Physiology and Medicine: Second Edition 2013 Published by Elsevier 545 564 doi:10.1016/B978-0-12-411557-6.00023-9

[B21] Jayanthi S Nilgiriwala KS Del Vecchio D Retroactivity controls the temporal dynamics of gene transcription ACS Synth Biol 2013 2 8 431 441 10.1021/sb300098w 23654274

[B22] Mutalik VK Guimaraes JC Cambray G Lam C Christoffersen MJ Mai QA Precise and reliable gene expression via standard transcription and translation initiation elements Nat Methods 2013 10 4 354 360 10.1038/nmeth.2404 23474465

[B23] Li B You L Predictive power of cell-to-cell variability Quantitative Biology 2013 1 2 131 139 10.1007/s40484-013-0013-3

[B24] Cambray G Guimaraes JC Mutalik VK Lam C Mai QA Thimmaiah T Measurement and modeling of intrinsic transcription terminators Nucleic Acids Res 2013 41 9 5139 5148 10.1093/nar/gkt163 23511967 PMC3643576

[B25] Salis HM The ribosome binding site calculator Methods Enzymol 2011 498 19 42 21601672 10.1016/B978-0-12-385120-8.00002-4

[B26] Elowitz MB Levine AJ Siggia ED Swain PS Stochastic gene expression in a single cell Science 2002 297 5584 1183 1186 10.1126/science.1070919 12183631

[B27] Raser JM O'Shea EK Noise in gene expression: origins, consequences, and control Science 2005 309 5743 2010 2013 10.1126/science.1105891 16179466 PMC1360161

[B28] Swain PS Elowitz MB Siggia ED Intrinsic and extrinsic contributions to stochasticity in gene expression Proc Natl Acad Sci U S A 2002 99 20 12795 12800 10.1073/pnas.162041399 12237400 PMC130539

[B29] Thattai M van Oudenaarden A Intrinsic noise in gene regulatory networks Proc Natl Acad Sci U S A 2001 98 15 8614 8619 10.1073/pnas.151588598 11438714 PMC37484

[B30] Paulsson J Summing up the noise in gene networks Nature 2004 427 6973 415 418 10.1038/nature02257 14749823

[B31] Hooshangi S Thiberge S Weiss R Ultrasensitivity and noise propagation in a synthetic transcriptional cascade Proc Natl Acad Sci U S A 2005 102 10 3581 3586 10.1073/pnas.0408507102 15738412 PMC552778

[B32] Pedraza JM van Oudenaarden A Noise Propagation in Gene Networks Science 2005 307 5717 1965 1969 10.1126/science.1109090 15790857

[B33] Murphy KF Adams RM Wang X Balazsi G Collins JJ Tuning and controlling gene expression noise in synthetic gene networks Nucleic Acids Res 2010 38 8 2712 2726 10.1093/nar/gkq091 20211838 PMC2860118

[B34] Elowitz MB Leibler S A synthetic oscillatory network of transcriptional regulators Nature 2000 403 6767 335 338 10.1038/35002125 10659856

[B35] Stricker J Cookson S Bennett MR Mather WH Tsimring LS Hasty J A fast, robust and tunable synthetic gene oscillator Nature 2008 456 7221 516 519 10.1038/nature07389 18971928 PMC6791529

[B36] Dunlop MJ Cox RS Levine JH Murray RM Elowitz MB Regulatory activity revealed by dynamic correlations in gene expression noise Nat Genet 2008 40 12 1493 1498 10.1038/ng.281 19029898 PMC2829635

[B37] Munsky B Neuert G van Oudenaarden A Using gene expression noise to understand gene regulation Science 2012 336 6078 183 187 10.1126/science.1216379 22499939 PMC3358231

[B38] Dublanche Y Michalodimitrakis K Kummerer N Foglierini M Serrano L Noise in transcription negative feedback loops: simulation and experimental analysis Mol Syst Biol 2006 2 41 16883354 10.1038/msb4100081 PMC1681513

[B39] Lou C Stanton B Chen YJ Munsky B Voigt CA Ribozyme-based insulator parts buffer synthetic circuits from genetic context Nat Biotechnol 2012 30 11 1137 1142 10.1038/nbt.2401 23034349 PMC3914141

[B40] Ang J Harris E Hussey BJ Kil R McMillen DR Tuning Response Curves for Synthetic Biology ACS Synth Biol 2013 2 10 547 567 10.1021/sb4000564 23905721 PMC3805330

[B41] Medema MH van Raaphorst R Takano E Breitling R Computational tools for the synthetic design of biochemical pathways Nat Rev Microbiol 2012 10 3 191 202 10.1038/nrmicro2717 22266781

[B42] Politi N Pasotti L Zucca S Casanova M Micoli G Cusella De Angelis MG Magni P Half-life measurements of chemical inducers for recombinant gene expression J Biol Eng 2014 8 1 5 10.1186/1754-1611-8-5 24485151 PMC3940292

[B43] Kelly JR Rubin AJ Davis JH Ajo-Franklin CM Cumbers J Czar MJ Measuring the activity of BioBrick promoters using an in vivo reference standard J Biol Eng 2009 3 4 10.1186/1754-1611-3-4 19298678 PMC2683166

[B44] Canton B Labno A Endy D Refinement and standardization of synthetic biological parts and devices Nat Biotechnol 2008 26 7 787 793 10.1038/nbt1413 18612302

[B45] Rosenfeld N Young JW Alon U Swain PS Elowitz MB Gene regulation at the single-cell level Science 2005 307 5717 1962 1965 10.1126/science.1106914 15790856

[B46] Braun D Basu S Weiss R Parameter estimation for two synthetic gene networks: a case study IEEE ICASSP 2005 5 769 772

[B47] Furusawa C Suzuki T Kashiwagi A Yomo T Kaneko K Ubiquity of log-normal distributions in intra-cellular reaction dynamics Biophysics 2005 1 25 31 10.2142/biophysics.1.25 PMC5036630 27857550

[B48] Van den Bulcke T Van Leemput K Naudts B van Remortel P Ma H Verschoren A SynTReN: a generator of synthetic gene expression data for design and analysis of structure learning algorithms BMC Bioinformatics 2006 26 7 43 16438721 10.1186/1471-2105-7-43 PMC1373604

[B49] Zucca S Pasotti L Politi N Cusella De Angelis MG Magni P A standard vector for the chromosomal integration and characterization of BioBrick™ parts in Escherichia coli J Biol Eng 2013 7 1 12 10.1186/1754-1611-7-12 23663425 PMC3662617

[B50] Kelly JR Tools and reference standards supporting the engineering and evolution of synthetic biological systems 2008 Ph.D. thesis, Massachusetts Institute of Technology

[B51] Anderson JC Voigt CA Arkin AP Environmental signal integration by a modular AND gate Mol Syst Biol 2007 3 133 17700541 10.1038/msb4100173 PMC1964800

[B52] Ozbudak EM Thattai M Lim HN Shraiman BI van Oudenaarden A Multistability in the lactose utilization network of Escherichia coli Nature 2004 427 6976 737 740 10.1038/nature02298 14973486

[B53] Sleight SC Bartley BA Lieviant JA Sauro HM Designing and engineering evolutionary robust genetic circuits J Biol Eng 2010 4 12 10.1186/1754-1611-4-12 21040586 PMC2991278

[B54] Pasotti L Zucca S Lupotto M Cusella De Angelis MG Magni P Characterization of a synthetic bacterial self-destruction device for programmed cell death and for recombinant proteins release J Biol Eng 2011 5 8 10.1186/1754-1611-5-8 21645422 PMC3127821

