# Supplementary material for: Modelling the effects of cell-to-cell variability on the output of interconnected gene networks in bacterial populations
Source: BMC Syst Biol. 2015 Jun 1;9(Suppl 3):S6. doi: 10.1186/1752-0509-9-S3-S6 (PMC4464218; doi:10.1186/1752-0509-9-S3-S6)
Supplement: Additional file 1 — Supplementary figures, results and tables. [file 1752-0509-9-S3-S6-S1.docx]

## Supplementary Figures

##
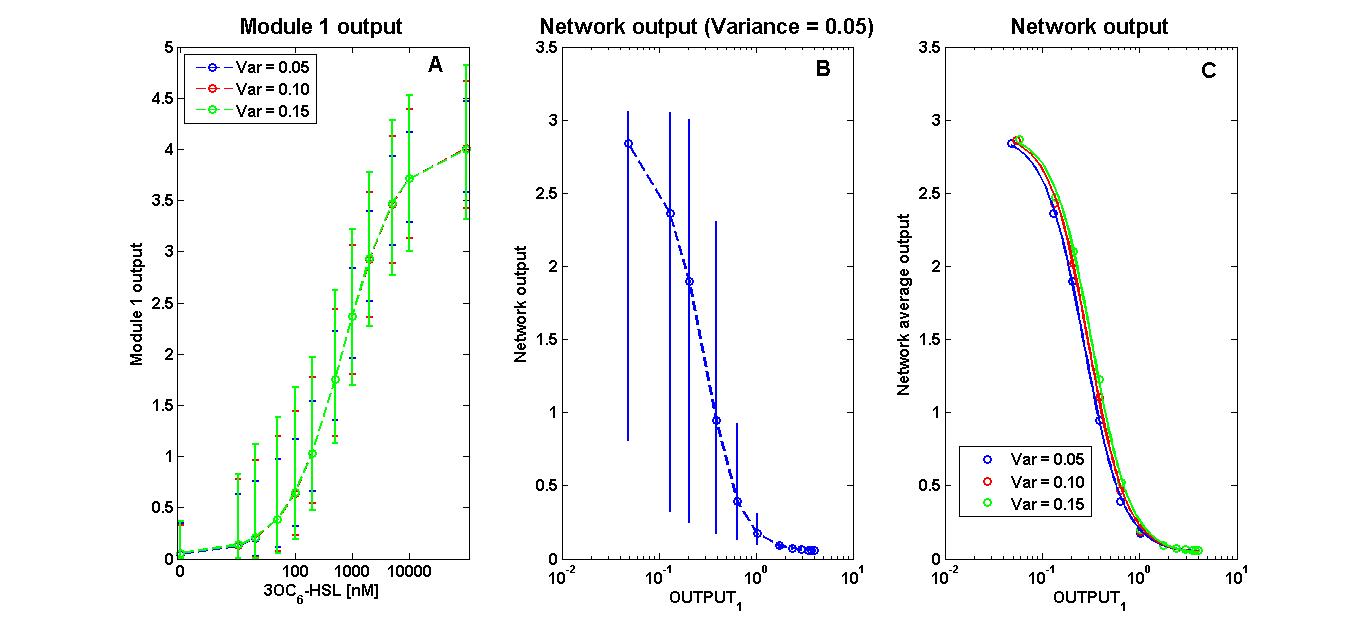


## Figure S1 - OUTPUT_1_ and OUTPUT_2_ signals in the two-module network (including the TetR/Ptet-based NOT gate) with constant VAR noise model.

A) OUTPUT_1_ signal for different noise entities, in response to 3OC_6_-HSL; in all the graphs, data points represent population-averaged values and error bars represent 95% confidence intervals. B) OUTPUT_2_ signal as a function of average OUTPUT_1_ in case of VAR=0.05. The average OUTPUT_1_ is computed from the 3OC_6_-HSL concentrations from panel A. Data points and error bars have the same meaning as above. Here, the cell-to-cell variability is derived from the propagation of noise from OUTPUT_1_. C) Population-averaged OUTPUT_2_ values as a function of average OUTPUT_1_. For all the VAR values, data are fitted with a Hill function (solid line). The estimated parameters are reported in Table 2 in the main text.

##
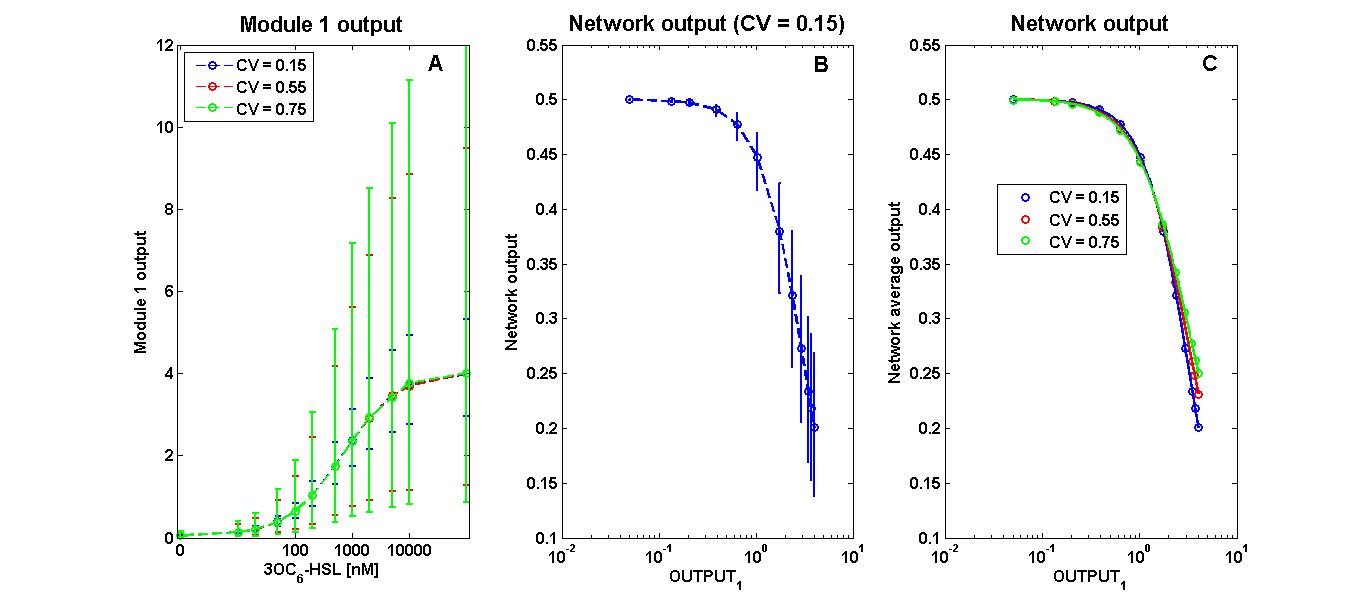


## Figure S2 - OUTPUT_1_ and OUTPUT_2_ signals in the two-module network (including the LacI/Plac-based NOT gate) with constant CV noise model.

A) OUTPUT_1_ signal for different noise entities, in response to 3OC_6_-HSL; in all the graphs, data points represent population-averaged values and error bars represent 95% confidence intervals. B) OUTPUT_2_ signal as a function of average OUTPUT_1_ in case of CV=0.15. The average OUTPUT_1_ is computed from the 3OC_6_-HSL concentrations from panel A. Data points and error bars have the same meaning as above. Here, the cell-to-cell variability is derived from the propagation of noise from OUTPUT_1_. C) Population-averaged OUTPUT_2_ values as a function of average OUTPUT_1_. For all the CV values, data are fitted with a Hill function (solid line). The estimated parameters are reported in Table 2 in the main text.

##
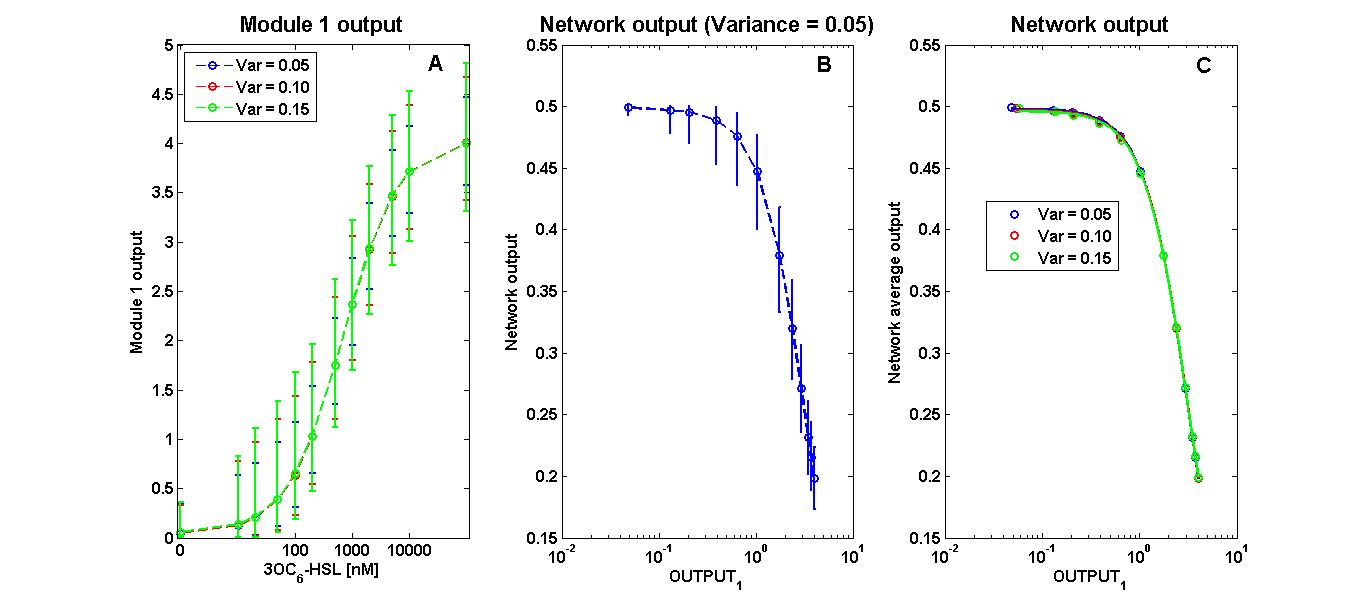


## Figure S3 - OUTPUT_1_ and OUTPUT_2_ signals in the two-module network (including the LacI/Plac-based NOT gate) with constant VAR noise model.

A) OUTPUT_1_ signal for different noise entities, in response to 3OC_6_-HSL; in all the graphs, data points represent population-averaged values and error bars represent 95% confidence intervals. B) OUTPUT_2_ signal as a function of average OUTPUT_1_ in case of VAR=0.05. The average OUTPUT_1_ is computed from the 3OC_6_-HSL concentrations from panel A. Data points and error bars have the same meaning as above. Here, the cell-to-cell variability is derived from the propagation of noise from OUTPUT_1_. C) Population-averaged OUTPUT_2_ values as a function of average OUTPUT_1_. For all the VAR values, data are fitted with a Hill function (solid line). The estimated parameters are reported in Table 2 in the main text.

##
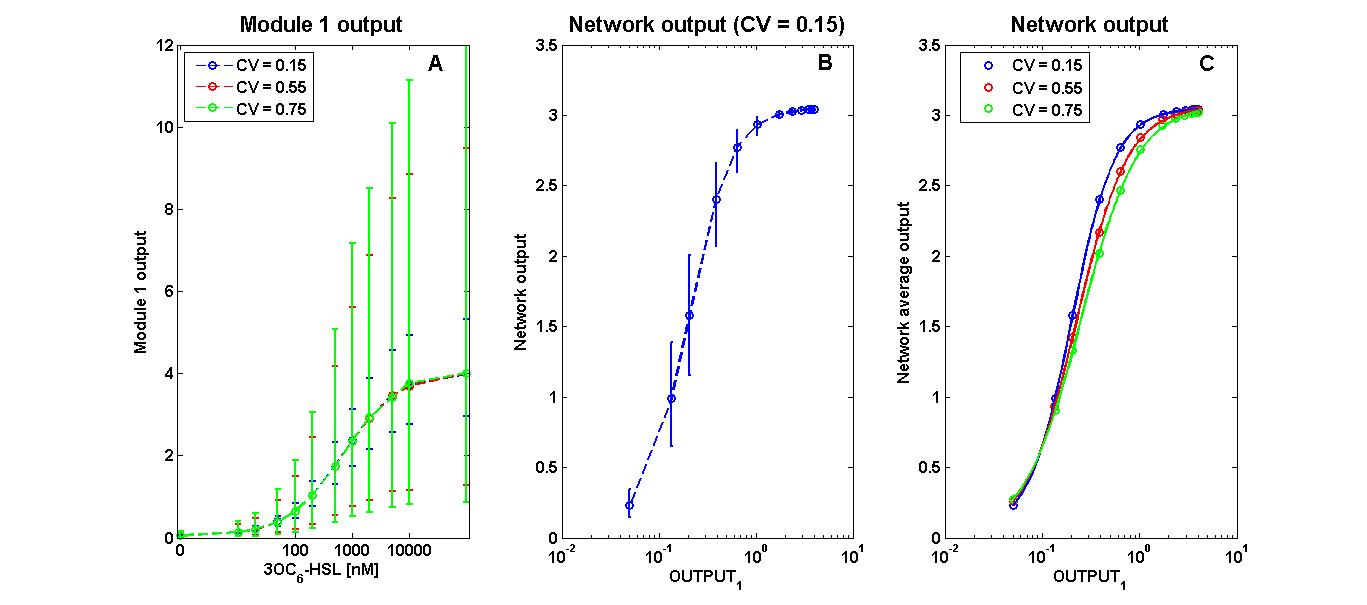


## Figure S4 - OUTPUT_1_ and OUTPUT_2_ signals in the two-module network (including the A/PA-based YES gate) with constant CV noise model.

A) OUTPUT_1_ signal for different noise entities, in response to 3OC_6_-HSL; in all the graphs, data points represent population-averaged values and error bars represent 95% confidence intervals. B) OUTPUT_2_ signal as a function of average OUTPUT_1_ in case of CV=0.15. The average OUTPUT_1_ is computed from the 3OC_6_-HSL concentrations from panel A. Data points and error bars have the same meaning as above. Here, the cell-to-cell variability is derived from the propagation of noise from OUTPUT_1_. C) Population-averaged OUTPUT_2_ values as a function of average OUTPUT_1_. For all the CV values, data are fitted with a Hill function (solid line). The estimated parameters are reported in Table 2 in the main text.

##
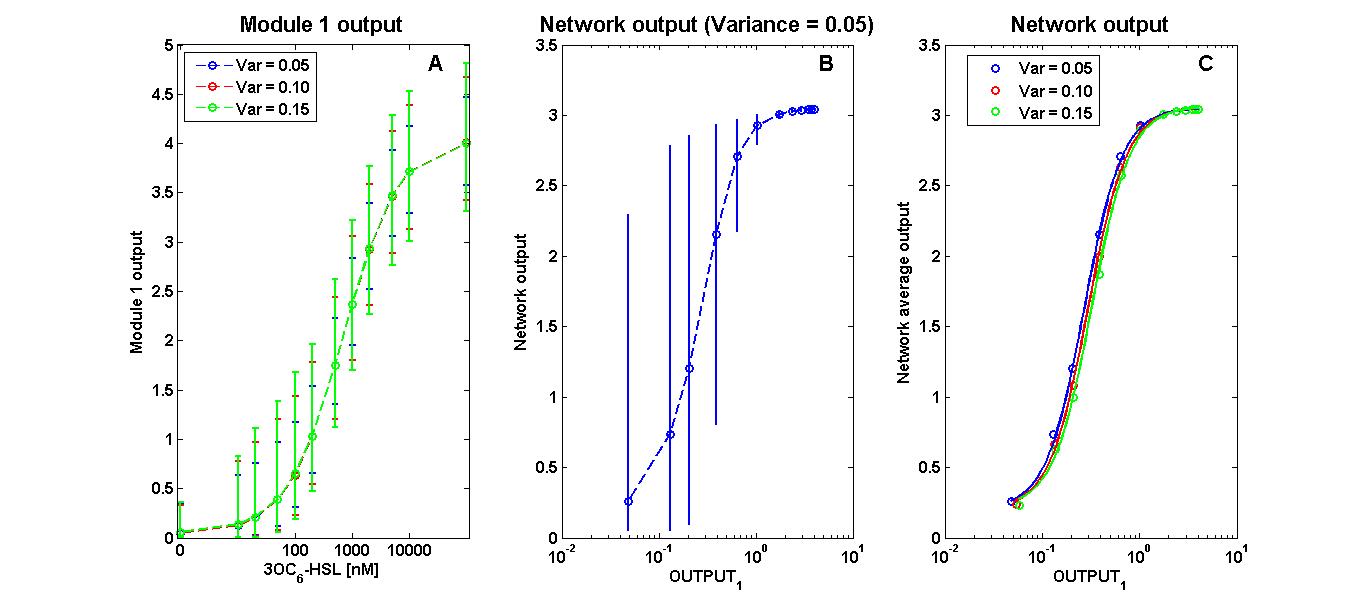


## Figure S5 - OUTPUT_1_ and OUTPUT_2_ signals in the two-module network (including the A/PA-based YES gate) with constant VAR noise model.

A) OUTPUT_1_ signal for different noise entities, in response to 3OC_6_-HSL; in all the graphs, data points represent population-averaged values and error bars represent 95% confidence intervals. B) OUTPUT_2_ signal as a function of average OUTPUT_1_ in case of VAR=0.05. The average OUTPUT_1_ is computed from the 3OC_6_-HSL concentrations from panel A. Data points and error bars have the same meaning as above. Here, the cell-to-cell variability is derived from the propagation of noise from OUTPUT_1_. C) Population-averaged OUTPUT_2_ values as a function of average OUTPUT_1_. For all the VAR values, data are fitted with a Hill function (solid line). The estimated parameters are reported in Table 2 in the main text.

##
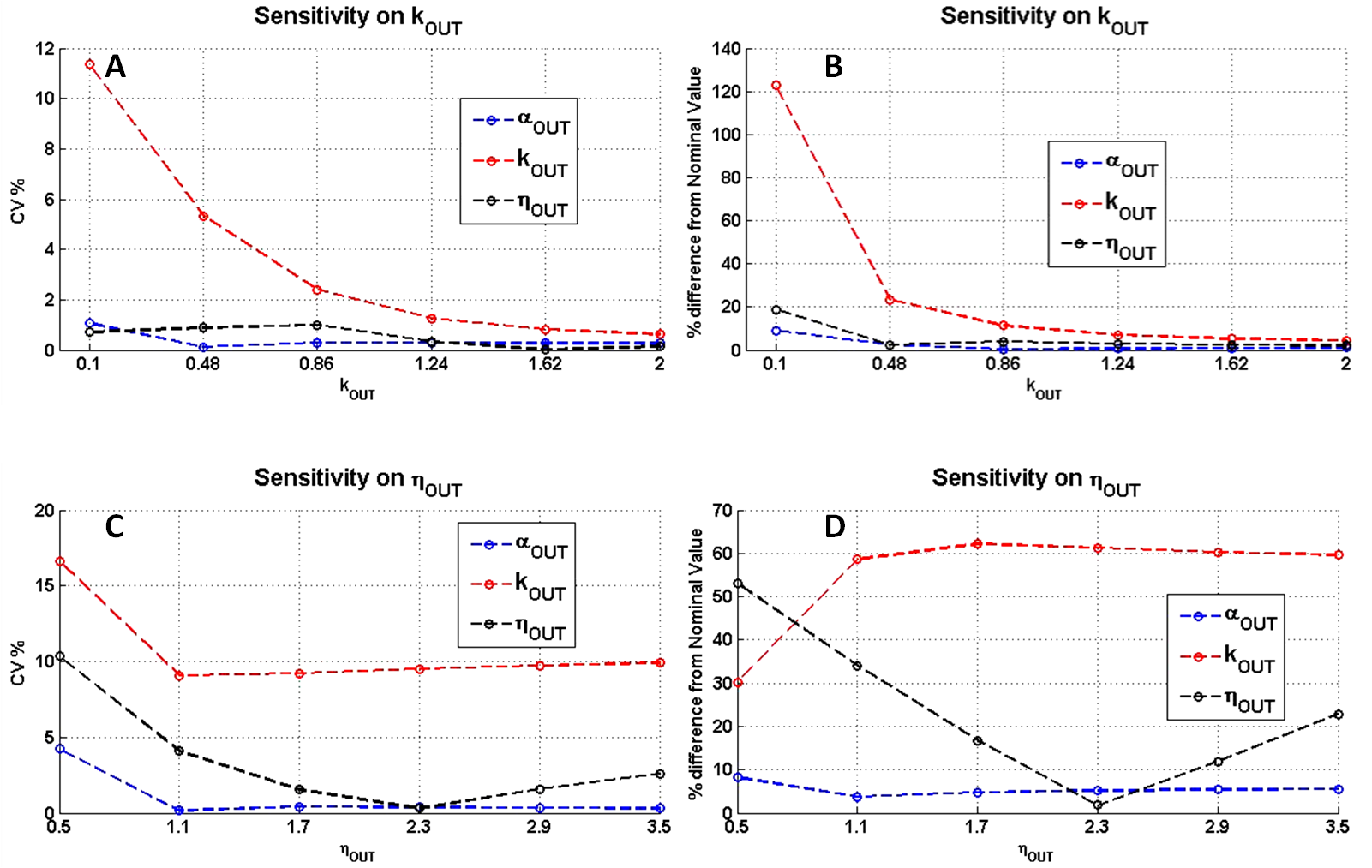


## Figure S6 - Sensitivity analysis for the two-module network with the TetR/Ptet-based NOT gate, when OUTPUT_1_ is affected by constant VAR noise: variability among the estimated parameters and maximum percent difference between estimated and true parameters.

## CV among the estimated parameters (A,C), and maximum percent difference between estimated and true parameters (B,D) for different values of (A-B) and $\boldsymbol{\eta}_{\boldsymbol{OUT}}$ (C-D). CV was computed among the parameters estimated for noise VAR=0.05, 0.1 and 0.15.


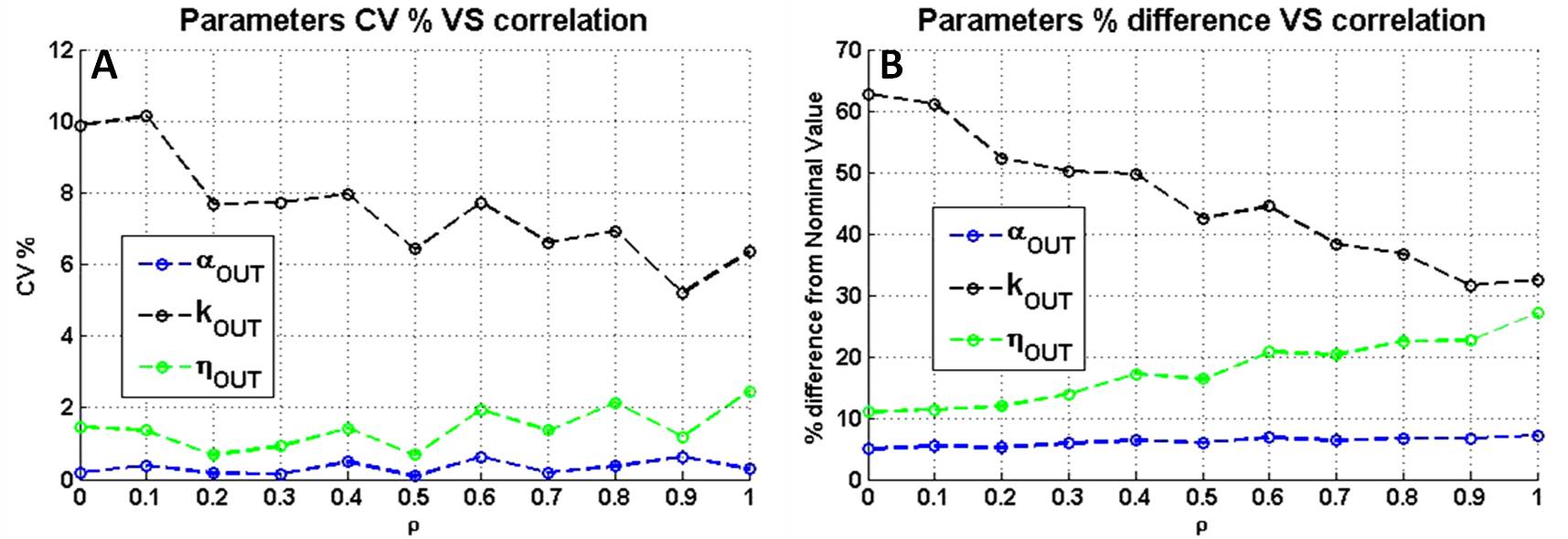


## Figure S7 - Analysis of the two-module network with the TetR/Ptet-based NOT gate, when OUTPUT_1_ and OUTPUT_2_ are affected by constant VAR noise with correlation coefficient ρ.

A) Variability among the estimated parameters, in terms of CV. B) Maximum percent difference between estimated and true parameter values. All the results are shown as a function of the correlation coefficient ρ, which is varied from 0 (no correlation) to 1 (maximum correlation). The increase of ρ value simulates an increase in proportion of the extrinsic component of noise over the total noise, which is composed by the intrinsic and extrinsic components.

##
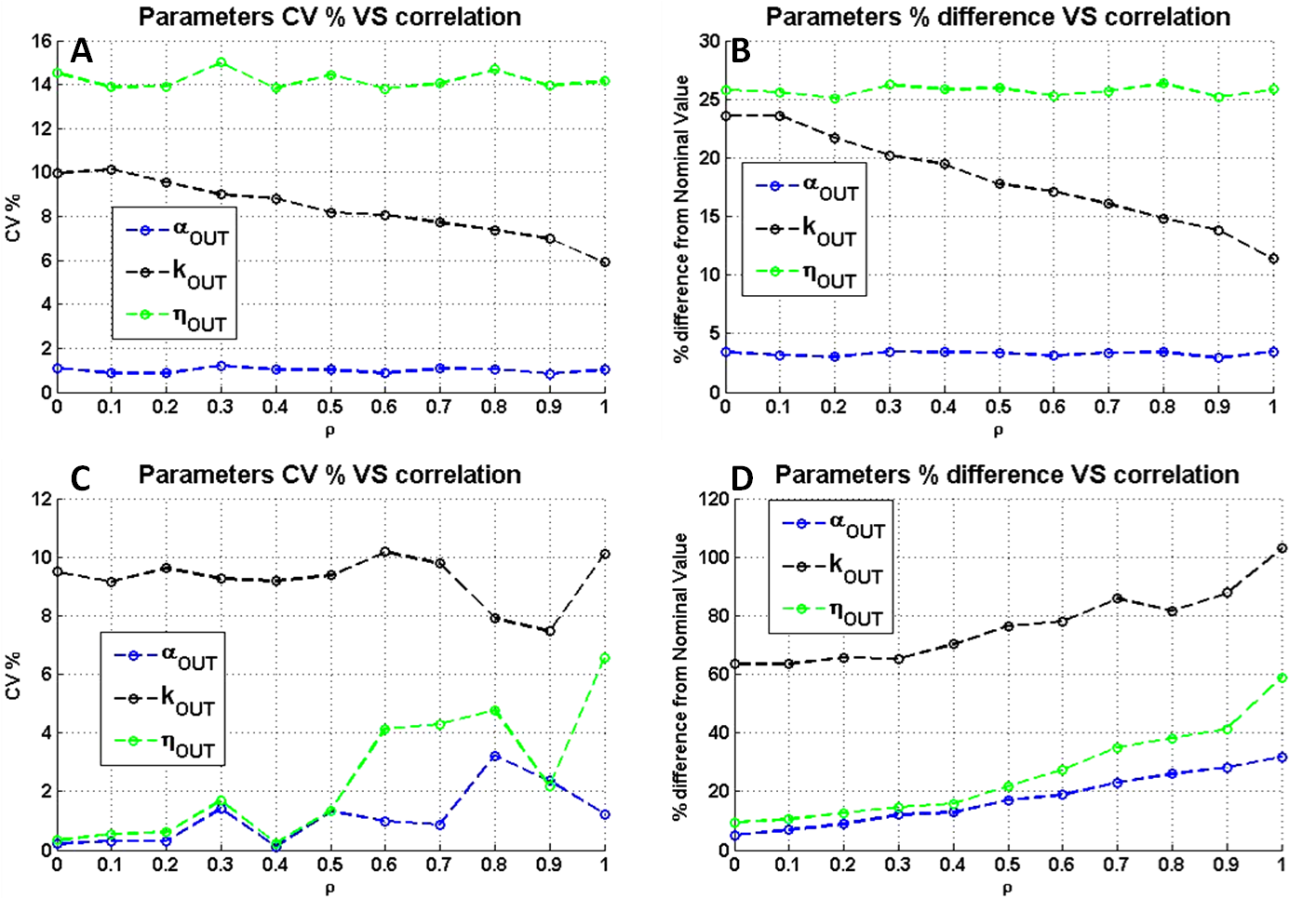


## Figure S8 - Analysis of the two-module network with the A/PA-based YES gate, when OUTPUT_1_ and OUTPUT_2_ are affected by constant CV or VAR noise with correlation coefficient ρ.

A, C) Variability among the estimated parameters, in terms of CV for the constant CV (A) and VAR (C) noise models. B, D) Maximum percent difference between estimated and true parameter values for the constant CV (B) and VAR (D) noise models. All the results are shown as a function of the correlation coefficient ρ, which is varied from 0 (no correlation) to 1 (maximum correlation). The increase of ρ value simulates an increase in proportion of the extrinsic component of noise over the total noise, which is composed by the intrinsic and extrinsic components.

##
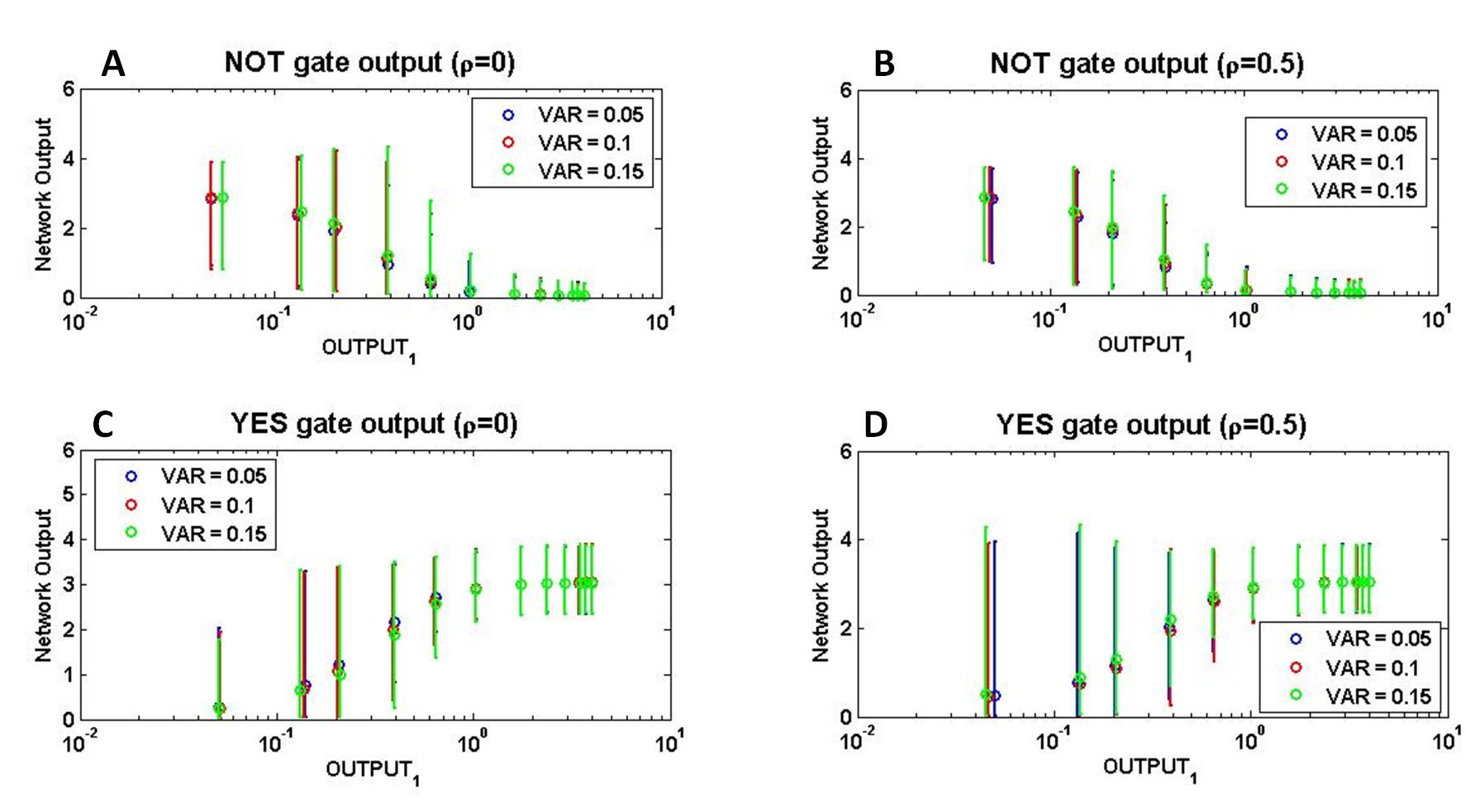


## Figure S9 - Cell-to-cell variability of OUTPUT_2_ as a function of OUTPUT_1_ in an *in silico* experiment involving a two-module network with the TetR/Ptet-based NOT gate or the A/PA-based YES gate, where constant VAR noise is applied to both OUTPUT_1_ and OUTPUT_2_ with two different correlation coefficients ρ.

## OUTPUT_2_ as a function of average OUTPUT_1_ values for the TetR/Ptet-based NOT gate (A-B) and the A/PA-based YES gate (C-D) for ρ=0 (A, C) and ρ=0.5 (B, D). The noise VAR is indicated in the panels. Error bars represent the 95% confidence intervals and thus indicate cell-to-cell variability in a population. The OUTPUT_1_ grid is reported in Figure S1A.

##
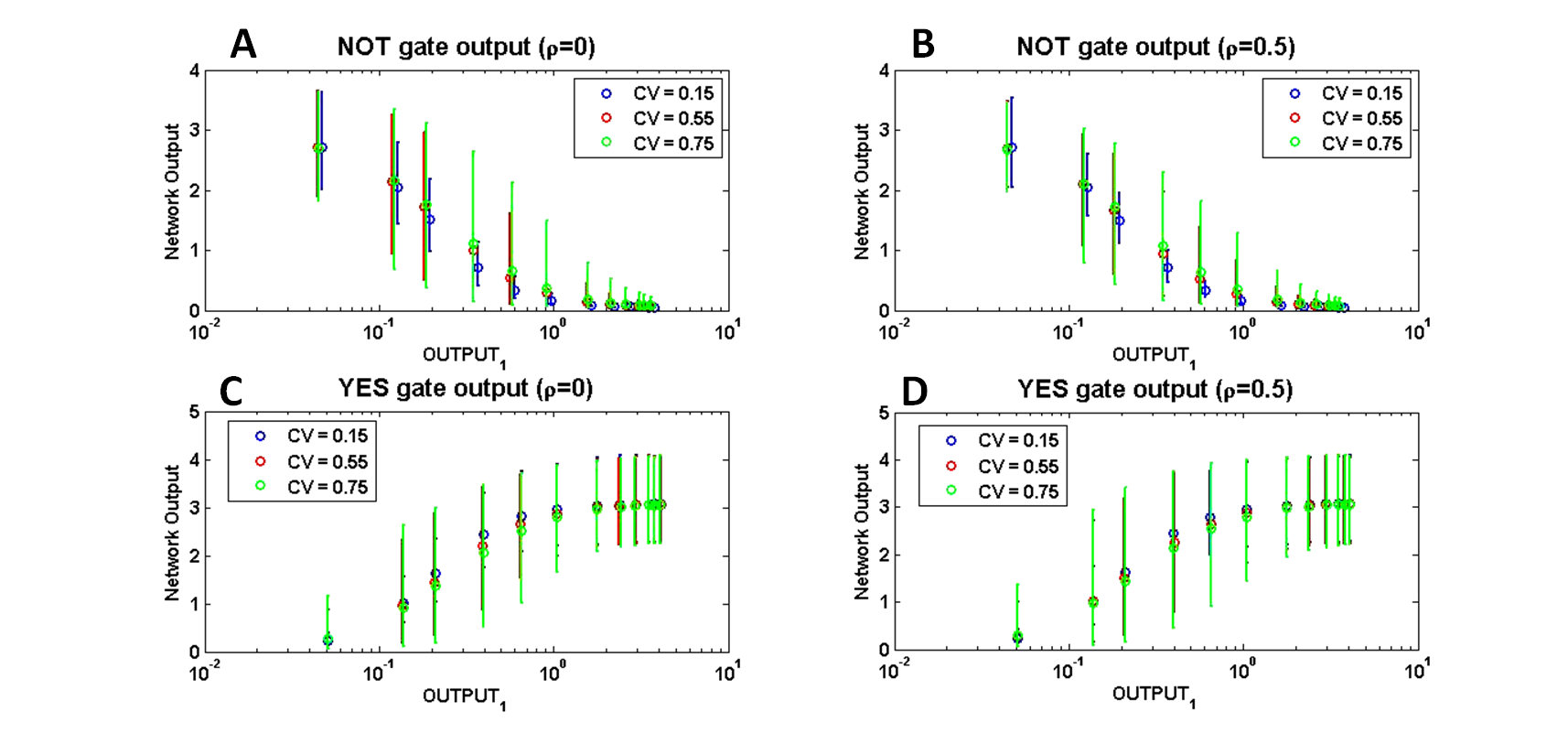


## Figure S10 - Cell-to-cell variability of OUTPUT_2_ as a function of OUTPUT_1_ in an *in silico* experiment involving a two-module network with the TetR/Ptet-based NOT gate or the A/PA-based YES gate, where constant CV noise is applied to both OUTPUT_1_ and OUTPUT_2_ with two different correlation coefficients ρ.

## OUTPUT_2_ as a function of average OUTPUT_1_ values for the TetR/Ptet-based NOT gate (A-B) and the A/PA-based YES gate (C-D) for ρ=0 (A, C) and ρ=0.5 (B, D). The noise CV is indicated in the panels. Error bars represent the 95% confidence intervals and thus indicate cell-to-cell variability in a population. The OUTPUT_1_ grid is reported in Figure S1A.

## Supplementary Results

## Analysis of the input-output function for a two-module interconnected network

Analogously to the analysis performed in the main text on a three-module network (see “Input-output function identification for an interconnected network” section), the input-output function of a two-module interconnected network (see Figure 1A in the main text) is also analyzed and identified as a black-box, whose behaviour is described by the Hill equation (Equation S1):

${OUTPUT}_{2}=\delta_{OUT}^{*}+\frac{\alpha_{OUT}^{*}}{1+\left( \frac{3OC_{6}-HSL}{k_{OUT}^{*}} \right)^{\eta_{OUT}^{*}}}$ (S1)

where 3OC_6_-HSL is the input, OUTPUT_2_ is the output of the whole network and parameters $\alpha_{OUT}^{*}$, $\delta_{OUT}^{*}$, $k_{OUT}^{*}$ and $\eta_{OUT}^{*}$ have the same meaning as in the Methods section.

Also in this case, we performed simulated experiments where: i) the transfer function of each single module was identified from population-averaged values and ii) the identified transfer functions were used to predict the black-box input-output function of the interconnected network. This process was repeated for each noise model and entity considered and Hill equation parameters were estimated for the black-box transfer function. The parameters reported in Table 1 were used to generate data, assuming the constant CV and VAR noise models, only applied to OUTPUT_1_ (see Figure 1D in the main text). The parameters describing the transfer function of individual modules were obtained previously (see Table 2 in the main text), while the estimated black-box function parameters are reported in Table S1 with their CV. These results depict that the resulting variability is very low, with the highest CV value for $k_{OUT}^{*}$ (17.9% and 12.2%) in the constant CV noise model and in the constant VAR model, respectively.

The TetR/Ptet-based NOT gate was also replaced by a YES gate with the same parameters and by a LacI/Plac-based NOT gate which represents a less sensitive switch compared to the TetR/Ptet system. The CV values obtained for the YES gate are identical to the ones of the NOT gate, again demonstrating that the logic of Module 2 does not give any contribution (data not shown). On the other hand, the CV values when using the LacI/Plac system are even lower than for the previous systems, resulting in a maximum CV of 9.3% and 0.8% ($k_{OUT}^{*}$ parameter) in the constant CV and VAR noise models, respectively (data not shown).

Overall, the results obtained for the two-module network are in accordance with the ones obtained for the three-module network, reported in the main text (see, for example, Table 3 in the main text).

As already discussed in the main text, the obtained parameters can be different from the ones estimated in a deterministic framework (reported in Table S2 for the network including the TetR/Ptet-based NOT gate), that is, without noise. For this reason, their maximum percent difference was computed. In both the constant CV and VAR noise models, the $k_{OUT}^{*}$ parameter is affected by the highest difference (46.6% and 88.7%, respectively), thus showing a relatively high deviation. Again, the results obtained for the two-module network are in accordance with the ones obtained for the three-module network, reported in the main text (which were 68.3% and 90.2%, respectively).

Considering this two-module network with the TetR/Ptet-based NOT gate and assuming a constant CV noise model, we performed a sensitivity analysis, where 6 parameters ($\alpha_{IN}$, $\delta_{IN}$, $k_{IN}$, $\eta_{IN}$, $k_{OUT}$, $\eta_{OUT}$) were individually varied; the CV among the estimated $\alpha_{OUT}^{*}$, $\delta_{OUT}^{*}$, $k_{OUT}^{*}$ and $\eta_{OUT}^{*}$ parameters was computed and the maximum percent difference with the parameters estimated in a deterministic framework was also calculated. The significantly marked trends in CV and percent differences are illustrated below.

The $\alpha_{IN}$ parameter, varied from 0.5 to 8, affected all the estimated parameters: $\alpha_{OUT}^{*}$ showed a modest decreasing trend for CV (from 8% to 2%, which remained constant for $\alpha_{IN}$>3) and for percent difference (from 18% to 2%, which remained constant for $\alpha_{IN}$>3); $k_{OUT}^{*}$ showed a increasing trend both for CV (from 6% to 17%) and for percent difference (from 15% to 45%), both saturating for $\alpha_{IN}$>2; $\eta_{OUT}^{*}$ showed a very moderate increasing trend, yielding CV values from 6% to 10% and percent difference values from 14% to 20%, both saturating for $\alpha_{IN}$>2.

The $\delta_{IN}$ (varied from 10^-5^ to 0.06) contributed to $k_{OUT}^{*}$ (CV from 12% to 20% and percent difference from 30% to 50%) and $\eta_{OUT}^{*}$ (CV from 14% to 10% and percent difference from 25% to 20%) only modestly.

In the tested value range for $k_{IN}$ (400-1000), no significant trends in CV and percent differences were observed.

The $\eta_{IN}$ parameter was varied between 0.5 and 3; the only estimated parameter affected by its variation is $k_{OUT}^{*}$, which shows a decreasing trend for CV (from 30% to 5%) and for percent difference (from 90% to 10%).

The $k_{OUT}$ parameter was varied from 0.1 to 2; it had a modest impact on $\alpha_{OUT}^{*}$ (CV from 0% to 10% and percent difference from 0% to 18%), while it had a larger impact on $k_{OUT}^{*}$, which showed a U-shaped dependency for CV (maximum CV of 24% and percent difference of 64%, both with a minimum of 0% when $k_{OUT}$ is 1.62).

Finally, the $\eta_{OUT}$ parameter (varied from 0.5 and 3.5) had an impact on $k_{OUT}^{*}$ (CV from 5% to 18% and percent difference from 10% to 45%, saturating for $\eta_{OUT}$>1.7) and on $\eta_{OUT}^{*}$ (CV from 0% to 24% and percent difference from 0% to 40%).

## Table S1 - Estimated parameters for the two-module network considered as a black-box function, for different noise models and entities, when the function is predicted from individual transfer functions derived from central tendency measures.

| **Parameter:** | $\boldsymbol{\alpha}_{\boldsymbol{OUT}}^{\boldsymbol{*}}$  **[RPU]** | $\boldsymbol{\delta}_{\boldsymbol{OUT}}^{\boldsymbol{*}}$  **[RPU]** | $\boldsymbol{k}_{\boldsymbol{OUT}}^{\boldsymbol{*}}$  **[nM]** | $\boldsymbol{\eta}_{\boldsymbol{OUT}}^{\boldsymbol{*}}$  **[-]** |
| --- | --- | --- | --- | --- |
| **constant CV** | 2.81  2.78  2.75  (1%) | 0.06  0.06  0.07 | 20.84  25.57  29.96  (17.9%) | 1.39  1.22  1.14  (10.5%) |
| **constant VAR** | 2.77  2.79  2.79  (0.4%) | 0.05  0.05  0.05 | 30.20  34.30  38.55  (12.2%) | 1.56  1.55  1.56  (0.5%) |

Parameters are obtained by fitting population-averaged values of OUTPUT_2_ as a function of 3OC_6_-HSL for different noise models and entity, applied to OUTPUT_1_. The three values reported in each cell correspond to CV=0.15, 0.55, 0.75 (for constant CV models) and to VAR=0.05, 0.1, 0.15 (for constant VAR models). The CV among the estimated parameters is reported in brackets.

## Table S2 - Estimated parameters for the two-module network considered as a black-box function, without noise affecting the network.

| $\boldsymbol{\alpha}_{\boldsymbol{OUT}}^{\boldsymbol{*}}$  **[RPU]** | $\boldsymbol{\delta}_{\boldsymbol{OUT}}^{\boldsymbol{*}}$  **[RPU]** | $\boldsymbol{k}_{\boldsymbol{OUT}}^{\boldsymbol{*}}$  **[nM]** | $\boldsymbol{\eta}_{\boldsymbol{OUT}}^{\boldsymbol{*}}$  **[-]** |
| --- | --- | --- | --- |
| 2.81 | 0.06 | 20.43 | 1.42 |

Parameters are obtained by fitting deterministic values of OUTPUT_2_ as a function of 3OC_6_-HSL.
